# Diamond nanopillar arrays for quantum microscopy of neuronal signals

**DOI:** 10.1117/1.NPh.7.3.035002

**Published:** 2020-08-06

**Authors:** Liam Hanlon, Vini Gautam, James D. A. Wood, Prithvi Reddy, Michael S. J. Barson, Marika Niihori, Alexander R. J. Silalahi, Ben Corry, Jörg Wrachtrup, Matthew J. Sellars, Vincent R. Daria, Patrick Maletinsky, Gregory J. Stuart, Marcus W. Doherty

**Affiliations:** a Australian National University, Research School of Physics and Engineering, Laser Physics Centre, Canberra, ACT, Australia; b Australian National University, John Curtin School of Medical Research, Eccles Institute of Neuroscience, Canberra, ACT, Australia; c University of Basel, Department of Physics, Basel, Switzerland; d Australian National University, Research School of Biology, Canberra, ACT, Australia; e University of Stuttgart, 3rd Institute of Physics, Stuttgart Research Centre of Photonic Engineering (SCoPE), Stuttgart, Germany

**Keywords:** nanopillars, nitrogen-vacancy, neuroimaging, neuromodeling, neurons

## Abstract

**Significance:** Wide-field measurement of cellular membrane dynamics with high spatiotemporal resolution can facilitate analysis of the computing properties of neuronal circuits. Quantum microscopy using a nitrogen-vacancy (NV) center is a promising technique to achieve this goal.

**Aim:** We propose a proof-of-principle approach to NV-based neuron functional imaging.

**Approach:** This goal is achieved by engineering NV quantum sensors in diamond nanopillar arrays and switching their sensing mode to detect the changes in the electric fields instead of the magnetic fields, which has the potential to greatly improve signal detection. Apart from containing the NV quantum sensors, nanopillars also function as waveguides, delivering the excitation/emission light to improve sensitivity. The nanopillars also improve the amplitude of the neuron electric field sensed by the NV by removing screening charges. When the nanopillar array is used as a cell niche, it acts as a cell scaffolds which makes the pillars function as biomechanical cues that facilitate the growth and formation of neuronal circuits. Based on these growth patterns, numerical modeling of the nanoelectromagnetics between the nanopillar and the neuron was also performed.

**Results:** The growth study showed that nanopillars with a 2-μm pitch and a 200-nm diameter show ideal growth patterns for nanopillar sensing. The modeling showed an electric field amplitude as high as $\approx 1.02\times 10^{10}\;\mathrm{mV}/m$ at an NV 100 nm from the membrane, a value almost 10 times the minimum field that the NV can detect.

**Conclusion:** This proof-of-concept study demonstrated unprecedented NV sensing potential for the functional imaging of mammalian neuron signals.

## 1 Introduction

Probing new frontiers in neuronal electrophysiology is the key to understanding how the brain works. It is becoming clearer that, to fully understand neuron excitability, there are a variety of requirements that must be met. These requirements can be presented in four major areas. The first is millisecond or submillisecond temporal resolution, which allows for the measurement of action potential (AP) changes over fast timescales.^1^ The second is nanoscale resolution across a field of view that is large enough to compass multiple neurons in a network.^1^ This enables the imaging of individual neuron compartments, such as dendritic spines.^2^ Third, the sensitivity of the probe must be exceptional, a sensor must be able to resolve millivolt changes in potential within submillisecond timescales to sense the smallest signals produced by neurons.^3^ Finally, the last major requirements are the compatibility and stability of the sensor, which should enable the sensor to probe a neuron without altering its behavior and make many measurements without the sensor failing or having toxic effects on the neuron. These requirements allow for imaging of neuron changes over a long period of time, which is important for the study of neuroplastic effects.^4^

There are a growing number of different techniques that can meet one or more of these requirements. Improvements in patch clamp techniques have pushed the use of cross-sectional nanoscale electrophysiology for investigating nanoscopic heterogeneities in ionic concentrations and local electric fields.^5^ Coupled with scanning ion conductance microscopy, it can perform wide-field imaging of neurons in a resting state.^2^ Voltage-sensitive fluorophores come in a variety of forms, some of which are able to image nanoscale structures, such as dendritic spines, with high sensitivity.^6^ However, no single device has the capacity to meet all of the listed requirements. The patch clamp technique can only measure APs at a single point on a neuron, removing the capacity of imaging propagation effects.^1^ Voltage-sensitive dyes can be difficult to use, requiring careful tailoring of the correct dye to a specific cell.^1^ In addition to this, any type of fluorophore that could be injected into a cell has an inherent time limit before photobleaching renders the sensor inoperable or phototoxicity kills the cell being imaged.^1^

Another often overlooked detail to consider when probing neurons at the nanoscale is how to best theoretically simulate and interpret neuron nanoelectromagnetics. Most spatial predictions of electromagnetics rely on variations of core conductor (CC) theory.^5^^,^^7^ The central assumption of this theory is that the density of ions inside and outside the neuron remains stationary during an AP. At micron distances from the membrane, where the ions can form a stable equilibrium, this assumption is true, making CC theory viable. At these length scales, the CC model has been experimentally verified by measuring the magnetic field of large neurons (200  μm in radius).^7^ However, at nanoscale distances from the membrane, ions flowing in and out of the neuron is precisely what generates the AP, making the assumption invalid.^5^

In this paper, we present a potential solution to nanoscale neurosensing with the application of a nitrogen-vacancy (NV) center.^8^^,^^9^ The NV is a defect in a diamond lattice consisting of a substitutional nitrogen atom with a nearest neighbor vacancy.^10^ The NV has unique spin-dependent photodynamics that allow its electronic spin to be optically initialized and read out. This allows for the NV’s electron spin resonance to be measured using a protocol known as optically detected magnetic resonance (ODMR). The ODMR resonances shift with perturbations from external electric^11^ and magnetic fields.^10^ Combining this with its atom-like size, the NV can perform nanoscale measurements of electric and magnetic fields.

The NV has been shown to have some of the best sensitivities and spatial resolutions for a room temperature sensor. Typical NV sensitivities are given as $891\;V/\mathrm{cm}\;{\mathrm{Hz}}^{-1/2}$ for DC electric fields^11^ and $40\;\mathrm{nT}\;{\mathrm{Hz}}^{-1/2}$^10^ for DC magnetic fields. These sensitivity values describe the smallest signal that the sensor can detect after a given acquisition time. The time duration of a peak neuron signal is around 1 ms;^12^ this means that the smallest field that an NV can detect within this time frame will be the sensitivity divided by the square root of the time duration. So for DC sensing, single NVs have been able to measure $(891\;V/\mathrm{cm}\;{\mathrm{Hz}}^{-1/2})/(\sqrt{10^{-3}}\;s)=2.8\times 10^{9}\;\mathrm{mV}/m$^11^ electric fields and $(40\;\mathrm{nT}\;{\mathrm{Hz}}^{-1/2})/(\sqrt{10^{-3}}\;s)=1.26\;\mu T$^10^ magnetic fields in a 1-ms acquisition time. In addition to its sensitivity, the NV has been shown to have submillisecond temporal resolution as well as spatial resolutions well into the nanometre scale.^13^ The NV itself is also a very stable atomic system that does not suffer from photobleaching, which allows for long-term imaging of a single sample.^8^ In addition to its physical capabilities, the NV is situated in a diamond structure. Diamond has been shown to be biologically compatible, having been successfully utilized in the past as a substrate to grow biological samples.^14^ They have also been proven to support functioning neurons via growth on unstructured nanodiamond monolayers that are assessed with calcium imaging.^15^ In fact, Barry et al.^8^ successfully measured neuronal signals along axons of marine fan worms by placing the axon on a flat diamond substrate with embedded NVs. In addition, work by Karaveli et al.^16^ demonstrated NV sensing of 20-mV changes in potential by utilizing it as a charge state sensor. However, as we move away from larger worm neurons toward the sensing of smaller mammalian neurons, the signal will also decrease beyond the sensitivity of the NV. It is this reduction in signal size that is the major barrier to NV neurosensing of mammalian neurons. Indeed, in this paper, our simulation results show that measuring magnetic signals of neurites 500 nm in radius^17^ is not possible using NVs in unstructured diamond.

**Fig. 1:**
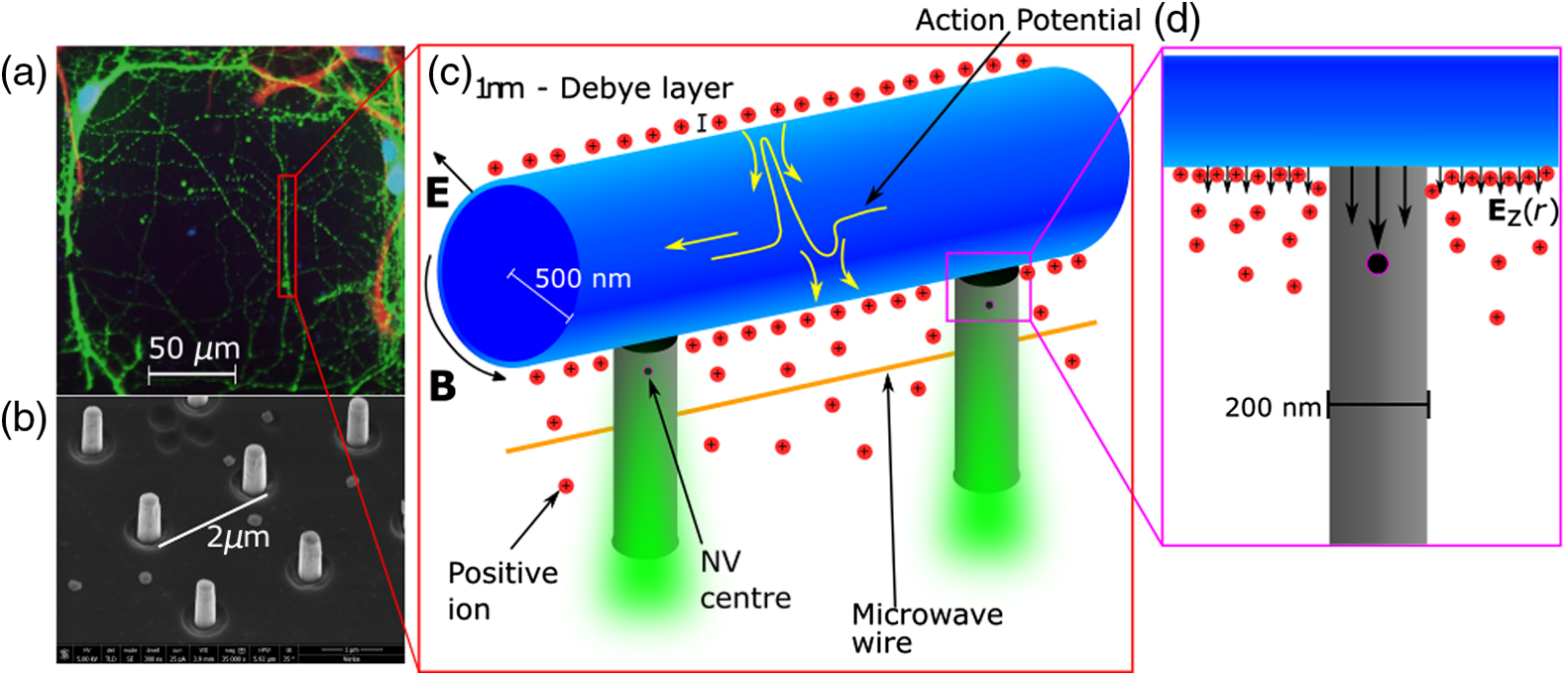
(a) Confocal image of the stained neurons (green) grown on a bed of nanopillars. The cell labeling was performed using immunofluorescent dyes for the neurons and astrocytes and a Hoechst stain for the cell nuclei. (b) Scanning electron microscope image of one diamond nanopillar geometry. (c) Cutout of a neurite grown on the pillars. The panel shows the cylindrical neurite with the surrounding positive ions that form a ≈1-nm thick Debye layer (negative ions not shown). Yellow arrows inside the cylinder indicate the current flow during an AP, which is depicted by the yellow line. The net ion charge and current densities generate the electric (E) and magnetic fields (B), respectively. These fields can be measured by the NVs situated in the gray diamond pillars. The sensing protocols use a green laser and microwaves to optically address the NV spin resonance. The pillars confine the laser light and direct the NV fluorescence. (d) Illustration of how the pillar removes the Debye screening charges by making contact with the neurite membrane. This increases the radial electric field at the position of the NV.

Our solution to this problem was inspired by work done with diamond nano-optics,^18^^–^^20^ as well as neuron growth studies on indium phosphate pillars by Gautam et al.^21^ We seek to sense neuron signals with the fabrication of diamond nanopillars, each with an NV sensor embedded within it. The pillar geometry yields three advantages. First, the shape of the pillars and the diamond’s high refractive index guide the excitation and emission light in and out of the diamond without significantly illuminating the neurons themselves^18^ [see Fig. 1(c)]. This light coupling phenomena improves the sensitivity of the NV by up to five times^18^ and reduces the phototoxic effect on the neurons from extended illumination. This will potentially increase the NV electric field sensitivity to $5.6\times 10^{8}\;\mathrm{mV}/m$ and the magnetic field sensitivity to 252 nT in the 1-ms acquisition time. The second advantage comes from the growth of the neurons on the pillars themselves. It has been demonstrated using other materials that pillar geometries act like a scaffold for neuron growth, guiding neurites in a single direction along the tips of the pillars^21^^,^^22^ [Figs. 1(a) and 1(c)]. This enhances the signal at the NV by coordinating neuron growth near the NVs themselves. In addition, the coordinated growth places a larger number of neurons in close proximity to the NVs. This provides more sites for experiment compared with an unstructured diamond. The third advantage lies in removing the Debye layer of the neuron; the key element that is absent in CC theories is the effects of the Debye screening layer.^5^ The Debye layer is the build-up of ions on either side of the membrane due to the electrochemical forces acting on individual ions.^12^ The Debye length is typically around 1 nm^12^^,^^23^ and is understood to greatly screen the electric potential external to the neuron,^5^^,^^12^^,^^24^ reducing its magnitude to zero over the course of a few nm. A diamond pillar placed in contact with the neuron could remove the screening ions, increasing the propagation of the external electromagnetic field [Fig. 1(d)]. It is our assumption that as long as the surface area where the nanopillar contacts the neuron membrane is small compared with the surface area of the enclosing cylindrical segment of the neuron, the removal of screening ions is unlikely to significantly alter the neuron’s natural function (refer to the Supplementary Material for more details). Based on the ${\mathrm{Ca}}^{2+}$ studies on unstructured diamond^15^ as well as ${\mathrm{Ca}}^{2+}$ studies performed on similar nanopillar arrays formed from other materials,^21^^,^^25^^–^^27^ we fully expect neurons to function normally on a diamond nanopillar array. However, ${\mathrm{Ca}}^{2+}$ studies on diamond nanopillar structures should be performed in future works to verify this.

In this paper, we first report a proof-of-concept study on the growth of neurons on a substrate of diamond nanopillars. The pillars are designed with a number of different diameters and pitches (distance from center to center of adjacent pillars) to assess which geometry is ideally suited for ordered neuron growth while maximizing photon collection efficiency for the sensing NVs. Second, we report simulations of the AP of a cylindrical axon with nanometer resolution. These simulations enable us to assess to the ability of NVs to detect the electric and magnetic fields associated with the AP as well as the enhancement of electric field detection offered by the nanopillar geometry. To the best of our knowledge, this will be the first study of neuron growth on diamond nanopillars and the first simulation of neuron electric and magnetic fields solved for an entire axon with nanoscale resolution. Similar simulations exist for the electric potentials of APs,^28^^,^^29^ but not for their electric and magnetic fields. The two key concepts of this work, nanopillar geometries and electric field sensing, provide a paradigm shift in NV neurosensing. The growth study experiment as well as the theoretical modeling provide the essential groundwork required to demonstrate this proof-of-concept work and allow for future research into real NV mammalian neurosensing.

## 2 Results

**Fig. 2:**
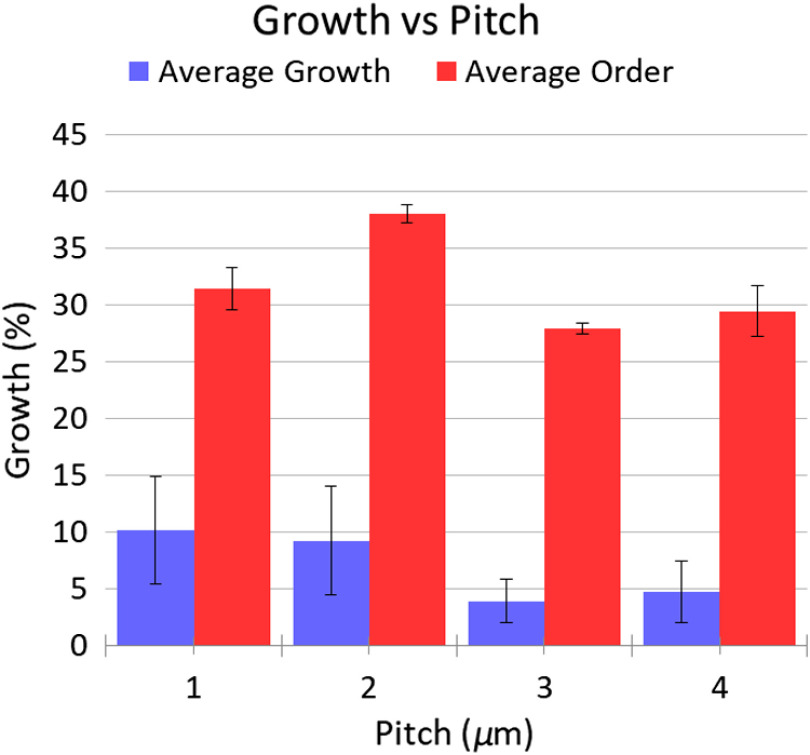
Table summary of growth as a function of pitch, averaged across all arrays of the same pitch. Error bars indicate one standard error of the sample mean. There is a general trend toward higher ordered growth for 2-μm pitch pillars.

Following the work of Gautam et al. and other neuron growth studies^21^^,^^22^^,^^30^ on indium phosphide (InP) substrates, we fabricated arrays of cylindrical diamond nanopillars (see Sec. 5). We then cultured neurons on top of them, staining them and analyzing their growth using fixed cell confocal microscopy. This confocal experiment is performed in a conventional upright setup in which the optical excitation and collection occur from above the neurons to directly focus on the neuron dyes and their relation to the pillar arrays. In the NV sensing experiment, the confocal microscopy will be performed in an inverted confocal setup with the laser emitting below the diamond substrate (∼0.3  mm in thickness) to take full advantage of the pillar waveguide effect. Each nanopillar array was 200×200  μm, separated by 400  μm, with a flat diamond between the arrays. This geometry was suitable for growing a small network of neurons on the top; however, much larger pillar arrays (centimeteres in area) are possible to support larger neuronal networks. The pillar pitch and diameter were varied between the arrays. The pitch was varied from 1 to 4  μm in 1  μm steps, and the pillar diameters were either 200 or 350 nm. In all arrays, the pillar height was 1  μm. Each unique array geometry was fabricated twice (totalling 16 arrays), and we labeled the arrays 0 to 15. Growth was prevented on arrays 0, 9, and 12 by air bubbles, so these arrays did not contribute to our results. Growth statistics were obtained for total growth and ordered growth (defined as being aligned with a pillar column or row) as functions of pitch, diameter, and fractional separation factor [(p−d)/p]. This statistical analysis was achieved by combining the results obtained from geometries with the same pitch, diameter, or fractional separation, respectively. Together, the total growth and the ordering metric allow for an understanding of which diamond nanopillar geometries produce the ideal growth for sensing studies. The ideal growth is the geometries that maximize the overall amount of neuron growth as well as the amount of neuron growth that is coordinated in close proximity to the NVs in the diamond pillars. Plots of the total and ordered growth versus pitch are shown in Fig. 2, and the remaining data can be found in the Supplementary Material.

A key observation is that all arrays showed non-negligible total growth. However, due to our small statistical sample size, the standard error is such that no single nanopillar geometry exhibited a statistically significant advantage for total growth. However, particular geometries had significantly larger growth ordering when considering pitch or fractional separation. These geometries had 2-μm pitch and either 200- or 350-nm diameters. When sampling for pitch, these geometries achieved 38% ordering on average with a standard error of ±0.8%. There was no significant dependence on pillar diameter. This is likely due to the small range of diameters that were sampled, which were chosen to approximately match the range of nanopillar diameters that maximizes optical collection efficiency.^18^^,^^19^

To model neuron electrophysiology, we consider an AP traveling along an infinitely long cylindrical axon. We assume that the AP is a nondispersive traveling wave that propagates with constant velocity. We further assume that the AP’s waveform is well described by the Hodgkin–Huxley (HH) model. The HH model is a well known and experimentally verified model of the transmembrane potential and ion flow.^31^ We then apply the coupled Poisson–Nernst–Planck (PNP) equations^28^^,^^29^^,^^32^ to solve for the coupled dynamics of the electric potential and ion concentrations within the neuronal media that are stimulated by the AP.

$$\epsilon \epsilon_{0}{\overset{\to }{\nabla }}^{2}V(r)=-\rho (r)=-e\overset{M}{\underset{i=1}{\sum }}z_{i}c_{i}(r),$$ (1)

$$\frac{\partial c_{i}}{\partial t}=-\overset{\to }{\nabla }\cdot \{D_{i}[\overset{\to }{\nabla }c_{i}+\mu_{i}c_{i}\overset{\to }{\nabla }V(r)]\},$$ (2)

where ϵ is the electric permittivity of the neuronal media, V(r) is the electric potential, ρ(r) is the charge density, $c_{i}(r)$ is the concentration of the i’th ion species with valence $z_{i}$ and diffusion constant $D_{i}$, M is the number of different ion species, $\mu_{i}=D_{i}^{2}k_{B}T/z_{i}e$ is the mobility of the i’th species, $k_{B}$ is Boltzmann’s constant, and T is the temperature. While this model can be expanded to include any number of different ion species, for ease of calculation, we only consider the monovalent species: sodium (${\mathrm{Na}}^{+}$), potassium ($K^{+}$), chlorine (${\mathrm{Cl}}^{-}$), and negatively charged proteins produced by the neuron (${\mathrm{OA}}^{-}$). Although other ions do exist, their concentrations are considered low enough to be neglected. In addition, the positive and the negative ions are respectively grouped into two effective species with diffusion constants defined by the average of those of the constituent species. The boundary conditions for the PNP at the axon membrane are defined by the transmembrane ion current and potential obtained from the HH equations. Once the PNP solutions are obtained, we calculate the electric field $\vec{E}$ via its quasi-DC relationship $\overset{\to }{E}=-\overset{\to }{\nabla }V$ with the electric potential, and the magnetic field $\vec{B}$ via Ampere’s law $\overset{\to }{\nabla }\times \overset{\to }{B}=\mu_{0}\overset{\to }{j}$ (where $\mu_{0}$ is the vacuum permeability) and the derived current density $\overset{\to }{j}=e\underset{i}{\sum }z_{i}\{D_{i}[\overset{\to }{\nabla }c_{i}+\mu_{i}c_{i}\overset{\to }{\nabla }V(r)]\}$. See Sec. 5 for further details of our simulation method and the Supplementary Material for a full derivation.

**Fig. 3:**
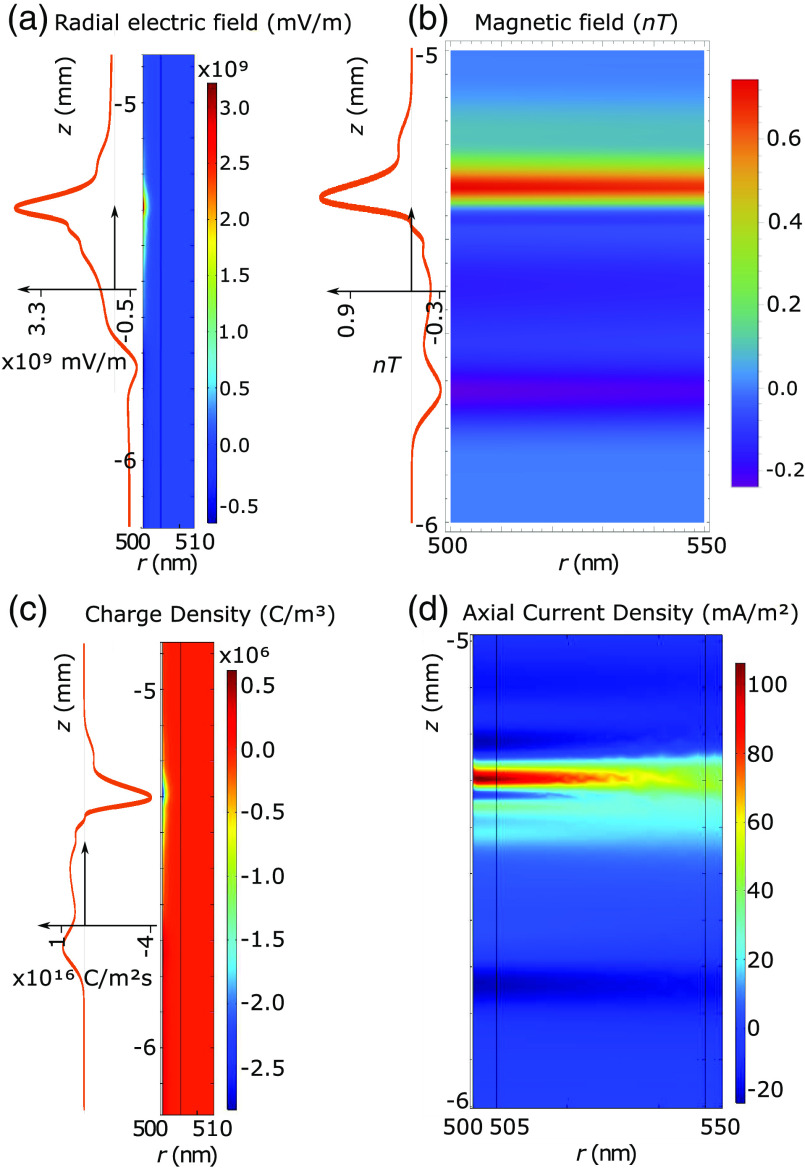
Simulation results for the (a) electric field, (b) magnetic field, (c) charge density, and (d) axial current density. On the left of (a)–(c), the electric field, magnetic field, and positive ion flux at the membrane are sketched, respectively, as orange lines, which are plotted in greater detail in Fig. S5 of the Supplementary Material (Sec. C). The membrane solutions are derived from experimentally verified HH equations. These solutions demonstrate how these quantities longitudinally propagate with the neuron signal.

**Fig. 4:**
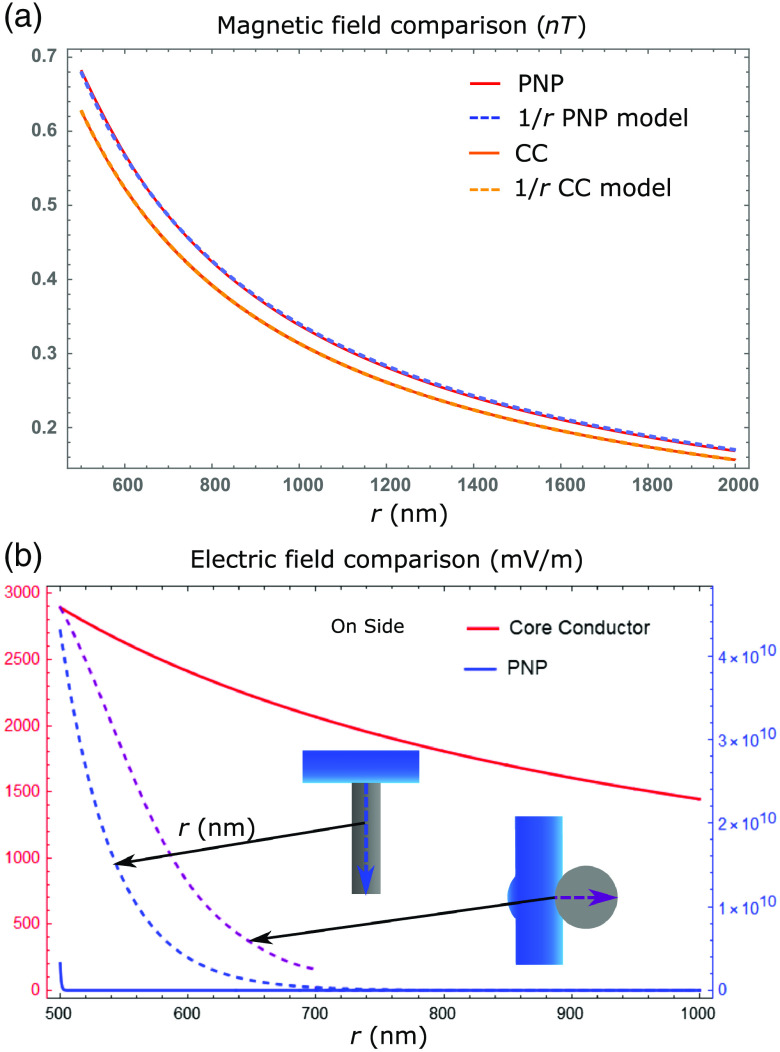
Radial one-dimensional plots of the (a) magnetic and (b) electric fields taken from the peak of the AP wave. The magnetic field plots feature the PNP solution and the CC solution derived from Woosley et al.^7^ as well as 1/r model fits for both. The electric field plots contain solutions to the PNP and CC models and the Laplace solutions for the electric field in the diamond when the neuron is in contact along the tip as well as along the side of the diamond pillar. The red axis is for the CC model and the blue axis is for the other three.

Surface plots of the simulation results are shown in Fig. 3. In the plots, the 500-nm mark corresponds to the external membrane radius. The orange lines are the traveling wave signal moving axially along the neuron. These lines are taken from the membrane boundary condition solutions for their respective quantities, which were derived from experimentally verified HH solutions. The orange line membrane solutions are plotted with more detail in Fig. S5 in the Supplementary Material (Sec. 5). There are also radial line graphs of the solutions that depict the electric and magnetic fields at the peak of the AP wave as well as 1/r model fits for the magnetic fields (Fig. 4). These plots also feature a CC solution calculated from the equations presented by Woosley et al.^7^ but altered to match the parameters of the mammalian neuron considered in this study.

As mentioned, the key result of the PNP calculation that is absent in CC theory is the effects of the Debye screening layer.^5^ The Debye screening is most evident in the electric field and charge density solutions. In Fig. 3(a), the resting radial electric field at the membrane (i.e., where the AP wave has zero amplitude) is $\approx -0.5\times 10^{9}\;\mathrm{mV}/m$. At the peak of the AP wave, this electric field at the membrane is significantly different $\approx 3.3\times 10^{9}\;\mathrm{mV}/m$. However, despite this dramatic change at the membrane, the Debye screening reduces the change to zero over ∼3  nm [solid blue line in Fig. 4(b)]. Comparing our PNP and CC solutions, it is clear that the inclusion of Debye screening in the PNP model has resulted in not only a much larger electric field at the membrane but also a much more rapid radial decay of the electric field. This result is also similar in the charge density solutions [Fig. 3(c)], where almost everywhere outside the neuron, the ion concentrations approach their bulk values, creating a zero net charge density. However, within the Debye layer and AP wave, the charge density decreases to as low as $\approx -3\times 10^{6}\;C/m^{3}$ due to the positive charge being transferred from outside to inside the axon, thereby increasing the potential and electric fields. Away from the AP wave, the charge density increases slightly to $\approx 0.5\times 10^{6}\;C/m^{3}$, reforming the positive-charge Debye layer in response to the internal negative charge present during the neuron resting conditions.

Debye screening has little effect on the magnetic fields [Fig. 3(b)]. The current density external to the axon is aligned with the internal current density. Consequently, this external current density reinforces the magnetic field generated by the internal current density [Fig. 3(d)]. However, the external current density is extremely small compared with the internal current density ($\approx 100\;\mathrm{mA}/m^{2}$ at its peak). Thus, the total magnetic field enhancement is negligible. The net magnetic field has a 1/r decay in both the PNP and CC models, so the magnetic field clearly decays radially much more slowly than the electric field (the magnetic field extending to over ≈2  μm, compared with the ≈3  nm of the electric field). Figure 4(a) shows that there is only a small difference in magnitude between the magnetic fields of the PNP and CC models. The similar radial decay, however, demonstrates how the PNP model can match the CC model at micron scales where the CC model is experimentally verified.^7^ A notable outcome of the PNP results is that the axial current density is much smaller than the radial current density. This is in keeping with the radial current density being primarily responsible for the change in charge density that generates the AP.

Critically, the magnetic field reaches a maximum of 0.7 nT at the membrane boundary. Whether the calculation is done using a full PNP simulation or CC theory^7^ or even by approximating the neuron as a current carrying wire,^8^ the results tend to suggest that a mammalian neuron can only produce a magnetic field that is less than a nT in magnitude. This signal is too small to be detected by an NV within the millisecond timescale of the neuron signal.^10^ Even with the light collection improvement from the pillar geometry, a fivefold improvement in the magnetic field sensitivity still will not allow for mammalian neuron AP sensing.^18^ For electric field sensing, although the magnitude of the field is larger than the minimum detectable electric field, the difficulty lies in placing an NV within the Debye layer external to the membrane. The closest range that an NV can be placed to the surface of a diamond is ≈5  nm while maintaining reasonable coherence and stability.^33^ In the simplest picture, if the diamond tip is in contact with the neuron, at this distance the field will have decayed to zero, thereby suggesting that NV sensing of both the neuron electric and magnetic fields is impossible.

However, this reasoning is too simplistic and does not consider the effect of the diamond nanopillar on the screening charge and current densities. As shown above, the effects of external currents are small, so the presence of the pillar will have a negligible effect on the magnetic field. However, the screening charge has profound effects on the electric field. By removing the screening charge between the neuron and the pillar tip (via good contact) and accounting for the much lower dielectric screening in the diamond (ϵ≈6) compared with the surrounding water (ϵ≈80), we expect the electric field inside the pillar to be much larger.

To model this enhancement, we solved Laplace’s equation inside a 200-nm-diameter pillar that is in contact with a neuron, such that the Debye screening layer is removed from the contact area. This simulation was done for two different coordinations of the pillar and neuron. In one, the neuron is on top of the pillar and in contact with the pillar’s complete circular top surface, and in the other, the neuron is on the side of the pillar near its tip and has a square contact area of the same size.

Plots of selected results are shown in Fig. 4(b) (see Sec. 5 for more details about the modeling). Specifically, these results are for the electric field magnitude in the pillar along a line extending from the center of the contact area along/through the central axis of the pillar for the on-top (blue dashed line) and on-side (purple dashed line) coordination. The electric field of the on-top coordination has an analytic solution with an exponential decay determined by a decay constant of ∼4.8/d where *d* is the diameter of the nanopillar. An NV depth of ≈5  nm and a pillar diameter much larger than this depth implies that an electric field as high as $\approx 3.2\times 10^{9}\;\mathrm{mV}/m$ will occur at the NV. For the on-side coordination, the electric field is predicted to be larger than for the on-top coordination at larger distances. Indeed, the results show that at 100 nm away from the neuron membrane (i.e., the central axis of the pillar), electric fields as high as $\approx 1.02\times 10^{9}\;\mathrm{mV}/m$ will occur. The larger field that arises when the neuron is on-side is due to the curvature of the contact area. This curvature implies that the distance from a point in the pillar to a charge on the neuron surface is on average smaller than for the flat contact area when the neuron is on top. This leads to a larger electric field for the on-side coordination.

## 3 Discussion

Our growth study results are consistent with the previous results of Gautam et al.,^21^ who also observed that fractional separation is important to growth ordering. Our observation that growth ordering also significantly depends on pitch can be explained by our small range of pillar diameters, which means that pitch was the dominant parameter in determining fractional separation. Having concluded this, future work is required to determine statistically significant total growth factors as well as finer pitch values to improve ordered growth. In addition to this, ${\mathrm{Ca}}^{2+}$ imaging studies can help to determine neuronal activity on the diamond substrate.

Given the NV electric field sensitivity mentioned above and the geometries stated, the neuromodeling shows that the NV should easily be able to detect APs within the signal timescale when the neuron is in both on-top and on-side locations of a diamond pillar. However, it is important to note that only qualitative information can be drawn from this type of study. One thing that is not considered in this model is how internal charges will reorganize in response to the changing electric field that the pillar provides. Although electric field enhancement is still predicted, the magnitude of such an enhancement cannot be accurately defined without a full model of internal and external neuron solutions to observe how the charges respond to the presence of the pillar. It should be noted that these simulations are far from trivial as the scale of the neuron signal compared with the nanopillar makes approximations problematic and the presence of the nanopillar itself removes symmetry, making the full PNP calculations computationally challenging. These simulations will be one of the focus areas of future work.

The analysis of the model also highlights the importance of having good contact between the neuron and the nanopillar in the region of the NV and that the location of this contact influences the electric field at the NV center. Previous neuron growth studies show that neurons tend to grow toward the tip of a pillar and can form contact with either the top or side, with contact on the side being more common.^21^^,^^34^ Future growth studies should seek to confirm this in a diamond substrate. In our results, it was not possible to determine the vertical position of the neuron in relation to the pillars nor the level of contact between the neuron and the pillar. Super-resolution confocal microscopy or SEM studies should be performed in the future to examine the precise vertical position of the neurons. One proposed means of improving neuron to substrate contact is to utilize a peptide coating that has been demonstrated to promote close contact between neurons and a growth substrate. These coatings, known as “engulfment promoting peptides” (EPPs) have been utilized in the past to promote close contact to microelectrode arrays^25^^,^^26^ and can be quantitatively assessed using TEM. In addition to this, TEM could also prove useful in determining the thickness and porosity of the protein coating used to promote neuron adhesion to the substrate (EPP or the an ornithin/laminin mixture described in Sec. 5). In this study, the coating was assumed to be dense enough such that ions could not penetrate the coating and thin enough that its effect on the propagating field from the neuron was negligible. However, these assumptions were not proven in any way. To the authors knowledge, the thickness and density of the coatings are not known and should be proven experimentally to be accurately factored into future simulations.

## 4 Conclusion

NV sensing of neuron signals has the capacity to provide a wealth of new information toward the understanding of neuron signaling for mammalian neurons. Our simulation results are essential for understanding what to expect from neuron signals and have the potential to improve our understanding of how neurons function at the most fundamental level. The simulations also have broader applications for design and optimization of other neurosensing techniques. The interesting result from our modeling lies in what external fields are measurable by the NV. Our calculations indicate that mammalian neuron magnetic fields are too small to be detected by NVs within the timescale of the neuronal signal. However, electric field sensing is possible due to the large signal and the potential capacity of a nanopillar to remove the screening ions. The pillar geometry also improves NV optical collection efficiency and coordinates neuron growth to improve NV positioning with a neurite. Indeed, growth ordering up to 38% was demonstrated, which implies that a high proportion of the neurite length is in a promising position for sensing.

This proof-of-concept simulation and growth study indicate the need to perform further simulations of NV pillars in a full interior and exterior solution of neuron electromagnetics to confirm the results presented in this paper. A larger scale growth study will also help confirm the ideal pillar dimensions for total growth and growth ordering. In addition, further studies of the neurite’s position relative to the pillar as well as the level of contact are also required. These studies will help determine the positioning of NVs within the pillars that on average optimizes coordination with the neuron and thus neuron sensing.

## 5 Experimental Section

### 5.1 Growth Study

The diamond pillar fabrication was performed using inductively coupled plasma reactive ion etching techniques following a recipe reported elsewhere.^35^ The fixed cell neuron culturing and microscopy was also performed following a procedure elsewhere,^21^ with primary cell cultures obtained from hippocampal regions of the brain of rats 0 to 2 days old. The tissues were dissociated in 1-mL Hank’s balanced salt solution (HBSS) consisting of 200 u/mL papain, activated with 1-mM L-cystein, and stabilized with 0.5-mM EDTA. Dissociated cells were then triturated in 1-ml HBSS containing 1% bovine serum albumin and suspended in Dulbecco’s modified Earl’s medium supplemented with 10% fetal bovine serum, 1% glutamine, 1% penicillin–streptomycin, and 1% B-27 supplement. The cells were then plated on the precoated diamond substrate, which were coated with dual layers of ornithin (50  μg/mL) and laminin (5  μg/mL) in 24-well plates. Cells were typically plated at a density of 150,000  cells/mL and incubated at 37°C in 5% ${\mathrm{CO}}_{2}$. The cells were then grown for up to 21 days *in vitro* (21 DIV) upon being labeled with Hoechst stain for cell nuclei, Tuj1-Alexa anti-rabbit (abcam) for β-III-tubulin (neurons), and GFAP-Alexa anti-mouse (abcam) for astrocytes. The neuron culturing and microscopy was also performed following a procedure described elsewhere,^21^ the only change in this procedure was the extra imaging of the diamond Raman fluorescence used to identify different diamond geometries, which is detailed in the Supplementary Material.

### 5.2 Neuron Image Processing

Once the neuron fluorescence was isolated on a pillar array, region of interest processing was performed to obtain a metric for total neuron growth on the array. In addition to this, we modified an image processing algorithm to determine the fraction of neurites coordinated with the rows or columns of the array. For each pillar array, we produced a mask that identified the “skeleton” of each neurite. We then algorithmically traced the neurites in the mask and summed their alignment to a pillar column/ row.^36^ Thus, a metric for coordinated or “ordered” growth was obtained by analyzing the length of coordinated neurites against the total length of all neurites. The skeletonization process replaces neurites as well as cell bodies with lines to be measured. For this reason, it was unsuitable to use the algorithm as a metric for total growth as the method does not take into account the varying thickness of cell bodies or neurites. See the Supplementary Material for more detail and the image analysis software at Ref. 37.

### 5.3 Neuron Modeling

To solve the PNP equations, we use axisymmetry arguments to simplify the calculation, as well as a traveling wave assumption to reduce the time-dependent three-dimensional PNP equations to two-dimensional equations, which were solved using COMSOL Multiphysics. Boundary conditions must be clearly stated and studied for both the ion concentrations and the electric potential. Far from the neuron radially, the boundary conditions are straightforward as the electric potential must go to zero, and the ion concentrations must reflect this with a stable equilibrium. Axially, far from the AP along the neuron, we expect the ion concentrations and the electric potential to reach a constant equilibrium corresponding to the resting potential (−68  mV). Thus, the derivative of the potential and flux must be zero in this region. At the membrane, we can derive a Neumann boundary condition using Gauss’ law. In this derivation, the charge density is expressed in terms of the radial current and the transmembrane potential, both of which are obtained from the standard HH^29^^,^^31^^,^^38^ equations of neuron APs. To obtain the membrane magnetic field boundary condition, we apply Ampere’s law, where the axial current is derived from the HH equations. The full derivation of our model as well as parameter values can be found in the Supplementary Material.

### 5.4 Electric Fields in Diamond Pillars

The electric fields inside the diamond pillar with a neuron in contact with it were solved using Laplace’s equation. This is achieved with the assumption that the charge inside the neuron is unperturbed by the presence of the pillar and that Debye screening fixes the electric potential to be zero everywhere on the diamond surface where the neuron is not in contact. Note that this ignores the small region at the edge of the contact area where, within the Debye layer extending from the neuron surface, the potential is nonzero at the diamond surface. We expect this to be a good approximation as long as the contact area dimensions are much larger than the Debye length, but not so large that the contact with the pillar changes the function of the neuron. In the on-top coordination, the solution was revolved around the cylindrical diamond pillar and solved analytically. For the on-side coordination, the solution was done numerically using COMSOL Multiphysics. Both methods are explained in more detail in the Supplementary Material.

## References

[1] Peterka D. S. Takahashi H. Yuste R., “Imaging voltage in neurons,” Neuron 69(1), 9–21 (2011). NERNET 0896-6273 10.1016/j.neuron.2010.12.010 21220095 PMC3387979

[2] Novak P. et al., “Nanoscale-targeted patch-clamp recordings of functional presynaptic ion channels,” Neuron 79(6), 1067–1077 (2013). NERNET 0896-6273 10.1016/j.neuron.2013.07.012 24050398 PMC3781326

[3] Perron A. et al., “Second and third generation voltage-sensitive fluorescent proteins for monitoring membrane potential,” Front. Mol. Neurosci. 2, 1–8 (2009). 10.3389/neuro.02.005.2009 19623246 PMC2706653

[4] Massobrio P. et al., “*In vitro* studies of neuronal networks and synaptic plasticity in invertebrates and in mammals using multielectrode arrays,” Neural Plast. 2015, 196195 (2015). 10.1155/2015/196195 25866681 PMC4381683

[5] Savtchenko L. P. Poo M. M. Rusakov D. A., “Electrodiffusion phenomena in neuroscience: a neglected companion,” Nat. Rev. Neurosci. 18(10), 598–612 (2017). NRNAAN 1471-003X 10.1038/nrn.2017.101 28924257

[6] Popovic M. A. et al., “Electrical behaviour of dendritic spines as revealed by voltage imaging,” Nat. Commun. 6, 1–12 (2015). NCAOBW 2041-1723 10.1038/ncomms9436 PMC4594633 26436431

[7] Woosley J. K. Roth B. J. Wikswo J. P., “The magnetic field of a single axon: a volume conductor model,” Math. Biosci. 76(1), 1–36 (1985). MABIAR 0025-5564 10.1016/0025-5564(85)90044-6

[8] Barry J. F. et al., “Optical magnetic detection of single-neuron action potentials using quantum defects in diamond,” Proc. Natl. Acad. Sci. U. S. A. 113(49), 14133–14138 (2016). PNASA6 0027-8424 10.1073/pnas.1601513113 27911765 PMC5150388

[9] Hall L. T. Simpson D. A. Hollenberg L. C., “Nanoscale sensing and imaging in biology using the nitrogen-vacancy center in diamond,” MRS Bull. 38(2), 162–167 (2013). MRSBEA 0883-7694 10.1557/mrs.2013.24

[10] Rondin L. et al., “Magnetometry with nitrogen-vacancy centers in diamond,” Rep. Prog. Phys. 77(5), 056503 (2014). RPPHAG 0034-4885 10.1088/0034-4885/77/5/056503 24801494

[11] Dolde F. et al., “Electric-field sensing using single diamond spins,” Nat. Phys. 7(6), 459–463 (2011). NPAHAX 1745-2473 10.1038/nphys1969

[12] Hille B., Ion Channels of Excitable Membranes, Sinauer Associates Inc., Sunderland (2001).

[13] Taylor J. M. et al., “High-sensitivity diamond magnetometer with nanoscale resolution,” Nat. Phys. 4(10), 810–816 (2008). NPAHAX 1745-2473 10.1038/nphys1075

[14] May P. W. et al., “Spatially controlling neuronal adhesion on CVD diamond,” Diamond Relat. Mater. 23, 100–104 (2012). 10.1016/j.diamond.2012.01.023

[15] Thalhammer A. et al., “The use of nanodiamond monolayer coatings to promote the formation of functional neuronal networks,” Biomaterials 31(8), 2097–2104 (2010). BIMADU 0142-9612 10.1016/j.biomaterials.2009.11.109 20035997

[16] Karaveli S. et al., “Modulation of nitrogen vacancy charge state and fluorescence in nanodiamonds using electrochemical potential,” Proc. Natl. Acad. Sci. U. S. A. 113(15), 3938–3943 (2016). PNASA6 0027-8424 10.1073/pnas.1504451113 27035935 PMC4839455

[17] Liewald D. et al., “Distribution of axon diameters in cortical white matter: an electron-microscopic study on three human brains and a macaque,” Biol. Cybern. 108(5), 541–557 (2014). BICYAF 0340-1200 10.1007/s00422-014-0626-2 25142940 PMC4228120

[18] Momenzadeh S. A. et al., “Nanoengineered diamond waveguide as a robust bright platform for nanomagnetometry using shallow nitrogen vacancy centers,” Nano Lett. 15(1), 165–169 (2015). NALEFD 1530-6984 10.1021/nl503326t 25438091

[r19] Babinec T. M. et al., “A diamond nanowire single-photon source,” Nat. Nanotechnol. 5(3), 195–199 (2010). NNAABX 1748-3387 10.1038/nnano.2010.6 20154687

[20] McCloskey D. J. et al., “Enhanced widefield quantum sensing with nitrogen-vacancy ensembles using diamond nanopillar arrays,” ACS Appl. Mater. Interfaces 12, 13421–13427 (2020). 10.1021/acsami.9b19397 32100531

[21] Gautam V. et al., “Engineering highly interconnected neuronal networks on nanowire scaffolds,” Nano Lett. 17(6), 3369–3375 (2017). NALEFD 1530-6984 10.1021/acs.nanolett.6b05288 28437614

[22] Specht C. G. et al., “Ordered growth of neurons on diamond,” Biomaterials 25(18), 4073–4078 (2004). BIMADU 0142-9612 10.1016/j.biomaterials.2003.11.006 15046898

[23] Syková E. Nicholson C., “Diffusion in brain extracellular space,” Physiol. Rev. 88(4), 1277–1340 (2008). PHREA7 0031-9333 10.1152/physrev.00027.2007 18923183 PMC2785730

[24] Ridley B. K., Quantum Processes in Semiconductors, 3rd ed, Oxford University Press, Oxford (2013).

[25] Hai A. et al., “Spine-shaped gold protrusions improve the adherence and electrical coupling of neurons with the surface of micro-electronic devices,” J. R. Soc. Interface 6(41), 1153–1165 (2009).1742-5689 10.1098/rsif.2009.0087 19474080 PMC2817159

[r26] Hai A. Shappir J. Spira M. E., “In-cell recordings by extracellular microelectrodes,” Nat. Methods 7(3), 200–202 (2010).1548-7091 10.1038/nmeth.1420 20118930

[27] Spira M. E. Hai A., “Microelectrodes get on your nerves multi-electrode array technologies for neuroscience and cardiology,” Nat. Nanotechnol. 8(2), 83–94 (2013). NNAABX 1748-3387 10.1038/nnano.2012.265 23380931

[28] Pods J. Schönke J. Bastian P., “Electrodiffusion models of neurons and extracellular space using the Poisson–Nernst–Planck equations—numerical simulation of the intra- and extracellular potential for an axon model,” Biophys. J. 105(1), 242–254 (2013). BIOJAU 0006-3495 10.1016/j.bpj.2013.05.041 23823244 PMC3703912

[29] Lopreore C. L. et al., “Computational modeling of three-dimensional electrodiffusion in biological systems: application to the node of Ranvier,” Biophys. J. 95(6), 2624–2635 (2008). BIOJAU 0006-3495 10.1529/biophysj.108.132167 18556758 PMC2527256

[30] Franze K., “The mechanical control of nervous system development,” Development 140(15), 3069–3077 (2013). 10.1242/dev.079145 23861056

[31] Hodgkin A. L. Huxley A. F., “A quantitative description of membrane current and its application to conduction and excitation in nerve,” J. Physiol. 117(4), 500–544 (1952). JPHYA7 0022-3751 10.1113/jphysiol.1952.sp004764 12991237 PMC1392413

[32] Corry B. Kuyucak S. Chung S.-H., “Tests of continuum theories as models of ion channels. II. Poisson–Nernst–Planck theory versus Brownian dynamics,” Biophys. J. 78, 2364–2381 (2000). BIOJAU 0006-3495 10.1016/S0006-3495(00)76781-6 10777733 PMC1300826

[33] De Oliveira F. F. et al., “Tailoring spin defects in diamond by lattice charging,” Nat. Commun. 8, 15409 (2017). NCAOBW 2041-1723 10.1038/ncomms15409 28513581 PMC5442357

[34] Hanson L. et al., “Characterization of the cell-nanopillar interface by transmission electron microscopy,” Nano Lett. 12(11), 5815–5820 (2012). NALEFD 1530-6984 10.1021/nl303163y 23030066 PMC11793935

[35] Appel P. et al., “Fabrication of all diamond scanning probes for nanoscale magnetometry,” Rev. Sci. Instrum. 87(6), 063703 (2016). RSINAK 0034-6748 10.1063/1.4952953 27370455

[36] Lee T. Kashyap R. Chu C., “Building skeleton models via 3-D medial surface axis thinning algorithms,” Graphical Models Image Process. 56(6), 462–478 (1994). 10.1006/cgip.1994.1042

[37] Reddy P. Hanlon L. Doherty M. W., “Neuron-length-estimation,” https://github.com/pmRed/neuron-length-estimation (2018).

[38] Zandt B. J. et al., “Neural dynamics during anoxia and the ‘wave of death’,” PLoS One 6(7), e22127 (2011). POLNCL 1932-6203 10.1371/journal.pone.0022127 21779384 PMC3135620

