# Supplementary material for: Diamond nanopillar arrays for quantum microscopy of neuronal signals
Source: Neurophotonics. 2020 Aug 6;7(3):035002. doi: 10.1117/1.NPh.7.3.035002 (PMC7406893; doi:10.1117/1.NPh.7.3.035002)
Supplement: Supplementary file 1 [file NPh_007_035002_SD001.pdf]

1 Supplementary Information

1.1 Section A: Growth study

In this section, a general hypothesis is discussed for the neuron growth mechanism on a pillar substrate. The optical wavelengths used in the growth study microscopy are described, and a general description of the light collection efficiency provided by the diamond pillars is presented. Table 1, shows excitation and emission wavelengths used in the microscopy, table 2 shows the full results of the growth study, figure 1 shows the total and ordered growth as a function of averaged diameter and figure 2 shows the total and ordered growth as a function of averaged fractional separation factor ($(p - d)/p$).

Although the specific mechanism for the ordered growth is still unknown, a strong hypothesis is that ordered growth is centered around mechanosensitive structures in the neuron cytoskeleton.[30] During neuron growth on a protein layered substrate (e.g. laminin on a diamond pillar), neurons express proteins such as integrin molecules in all directions, which bind to the laminin in the extra-cellular matrix forming a new protein complex. This protein complex then binds to actin on the neuron cytoskeleton and mechanosensitive ion channels on the cytoskeleton initiate various mechanotransduction pathways, which encourages cellular growth at the point of the binding. As this process repeats itself, a regular line of pillar structures can encourage the process to continue in a line, thus producing directionally ordered growth. In this way, external forces on mechanically sensitive ion channels direct neuron growth in response to physical cues such as a diamond nanopillar.[30] One result of this is that there is a distance in which, ordered growth is maximized. The protein complexes produced in the growth process have a finite size, creating a range where the neuron is mechanosensitive. If the distance between the pillars is larger than this, then there

won't be a connection of growth between adjacent pillars. However if the distance is too small, then the neurons could potentially grow in any direction towards an adjacent pillar (e.g. diagonally instead of vertically or horizontally) as long as it is within the range produced by the protein complex, thus producing no ordered growth at all similar to a flat substrate. This necessitates the need for a growth study to find the ideal pillar geometry that matches the growth mechanism.

The dyes used for the growth study are listed in table 1. The table also includes the excitation and emission wavelengths for the various dyes as well as the Raman line we used to image the diamond itself. There was some overlap between the diamond fluorescence and the Glia dye, however as the diamond imaging was only used to find the pillar patch neurons were growing on, this did not affect the overall results. With the above exceptions, the cell labelling and confocal microscopy techniques were exactly the same as preformed in by Gautam et al.[21]

The pillar pitches chosen following work from Gautam et al.,[21] where distances between pillars were chosen to maximize the growth via the binding protein complexes. The diameters and heights were chosen by following work from Momenzadeh et al.[18] In their work, they calculated the size and shape of pillars which maximizes light collection efficiency for shallow NV implantation. The general principle is that the pillar acts like a waveguide, whose size and shape matches to fundamental HE (hybrid electric) modes, guiding light out below the pillar into the detection system. Pillars 1 μm in height with 200 and 350 nm diameters are ideally shaped to maximize the number of fundamental modes guided in and out of the pillar.

Section B: Neuron image processing

The total growth factor was performed by using ROI image processing in order to measure the growth of neurons as a fraction of the diamond pillar patch. Calculating ordered growth required

Table 1 Table of fluorescent components used in the confocal microscopy and the structures being imaged with them.

Technique	Type Imaged	Laser Excitation (nm)	Emission band (nm)	Reference
Tuj1-Alexa	Neurons	488	525/50	Gautam et al[21]
GFAP-Alexa	Glia (Astrocytes)	561	595/50	Gautam et al[21]
Hoechst stain	Cell nuclei	405	450/50	Gautam et al[21]
Raman	Diamond	561	606	-

Table 2 Table of the growth results, including patch label, total growth, ordered growth, fractional separation and volume ratios. Note that patches 0, 9 and 12 had problems with the growth, requiring their data results to be removed from the published results and analysis.

Patch	Pitch (p) (μm)	Diameter (d) (μm)	Distance ratio (p-d)/d	Volume ratio $\pi r^2/p^2$	Total Growth %Area	Total neurite T_i (μm)	Ordered neurite T_o (μm)	Ordered growth $\frac{T_i}{T_o}$(%)
0	1	0.2	0.8	0.031	0	0	0	0
1	1	0.2	0.8	0.031	15.9	3762.5	1116.4	29.7
2	1	0.35	0.65	0.096	13.7	5075.4	1494.5	29.4
3	1	0.35	0.65	0.096	0.7	331.7	116.3	35.1
4	2	0.2	0.9	0.008	12.8	3014.8	1148.4	38.1
5	2	0.2	0.9	0.008	21.1	4119	1593.3	38.7
6	2	0.35	0.825	0.024	2.2	796.4	315.0	39.6
7	2	0.35	0.825	0.024	0.8	475.1	169.9	35.8
8	3	0.2	0.933	0.003	0.3	341.8	95.2	27.9
9	3	0.2	0.933	0.003	0	0	0	0
10	3	0.35	0.833	0.011	6.9	1316.5	356.0	27.0
11	3	0.35	0.833	0.011	4.5	1503.7	433.2	28.8
12	4	0.2	0.95	0.002	0	0	0	0
13	4	0.2	0.95	0.002	10.1	1965.5	521.5	26.5
14	4	0.35	0.913	0.006	2.0	695.9	235.5	33.8
15	4	0.35	0.913	0.006	2.1	1052.5	294	27.9

the design of a specific algorithm. The orientation and length of each neuron can be determined by calculating the center line of each neurite. A binary mask showing neurite center lines was calculated by applying a skeletonisation algorithm.[36] This algorithm reduces every neurite and cell body in the image to a single pixel line without changing the overall structure of the image. The binary mask was convolved by a 3x3 kernel, such that neurite ends, midpoints, and intersections can be uniquely identified. Treating the resulting image as an undirected graph, we can parameterize each neurite by searching the graph for connected lines of pixels. Our search algorithm starts from

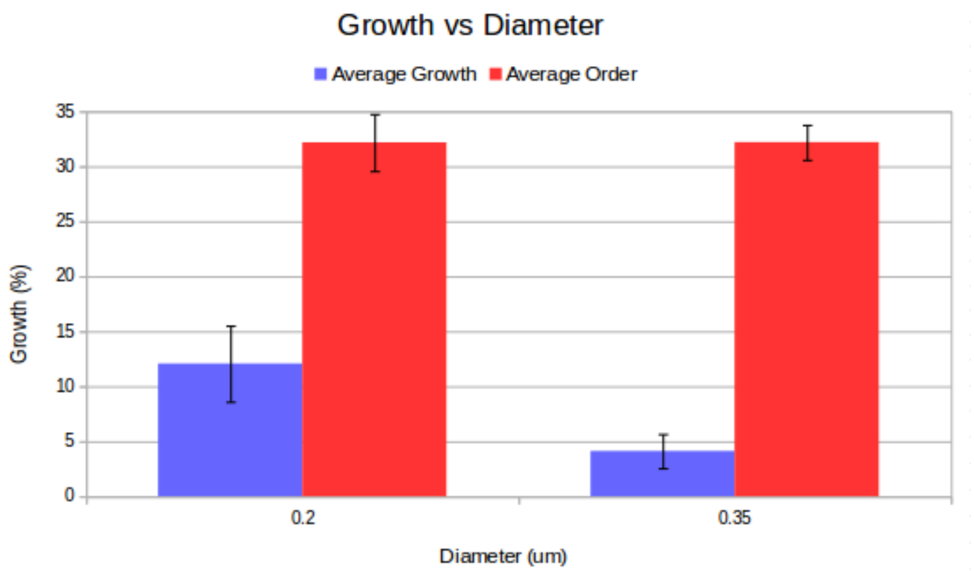

S 1 Table summary of growth as a function of diameter, averaged across all patches of the same diameters. Error bars indicate one standard error of the sample mean. There is no statistical dependence of diameter on ordered or total growth

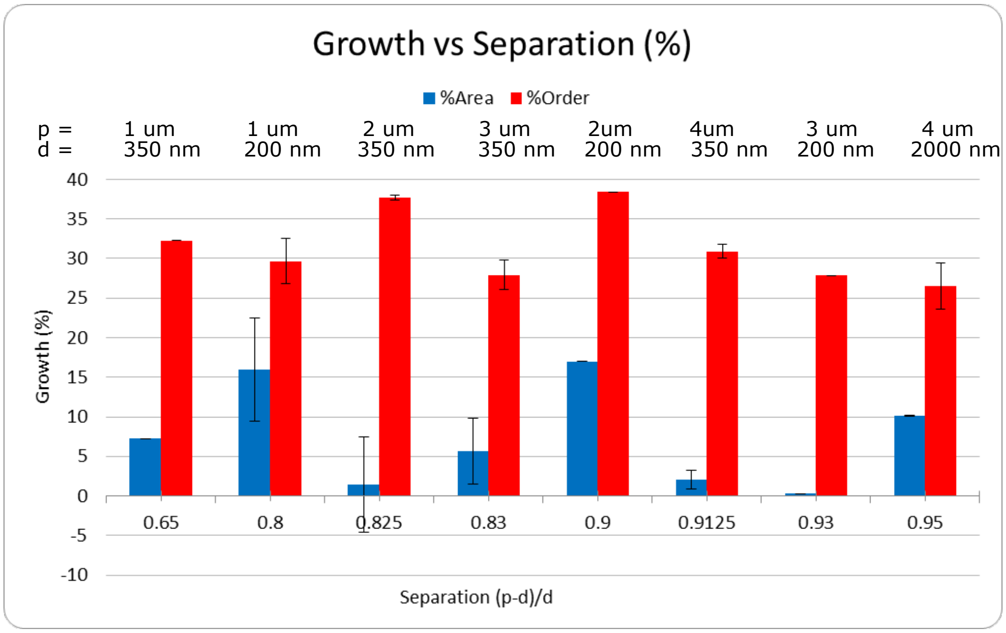

S 2 Table summary of growth as a function of the fractional separation, averaged across all patches of the same separation. The pitch and diameter for each separation is displayed above. Error bars indicate one standard error of the sample mean. The results show that high ordering occurs around 2 μm pitches which is a similar result when averaged over pitch alone.

any endpoint or intersection and traverses connected pixels until it finds another endpoint. Each set of connected pixels is called a path. This process is repeated for each endpoint until all paths

are identified. Each path parametrizes the centerline of a neurite. We perform the following line integral along these parameterized center lines to estimate how well the neurite aligns to a pillar line:

$$T_i = \sum_{i=1}^{L} \int_0^T |\frac{\partial}{\partial t}\vec{f_i}|dt \tag{3}$$

$$T_o = \sum_{i=1}^{L} \int_0^T \Theta(t)|\frac{\partial}{\partial t}\vec{f_i}|dt \tag{4}$$

where T_i is the total length of all the summed neurites (paths), T_o is the total length of the aligned (or ordered) neurites and $\vec{f_i}$ is the neurite's vector component parametrized by the length t:

$$\vec{f_i} = x(t)\hat{x} + y(t)\hat{y} \tag{5}$$

where the coordinate vectors $\hat{x}$ and $\hat{y}$ are chosen to coincide with the directions of the rows and columns of the nanopillar array.

The $\Theta(t)$ term represents a piece-wise function, which defines alignment by measuring the angle between the neurite vector component and the vector components of the pillar lines:

$$\Theta(t) = \begin{cases} 1, & arccos\left(\frac{\frac{\partial \vec{f}}{\partial t}\cdot\hat{u}}{|\frac{\partial \vec{f}}{\partial t}|}\right) \leq \frac{\pi}{36} \\ 0, & arccos\left(\frac{\frac{\partial \vec{f}}{\partial t}\cdot\hat{u}}{|\frac{\partial \vec{f}}{\partial t}|}\right) > \frac{\pi}{36} \end{cases}$$

where $\hat{u} = \hat{x}$ or $\hat{y}$. The principle is that the angle between a neurite vector components and a vertical ($\hat{y}$) or horizontal ($\hat{x}$) line of pillars is measured, if that angle is larger than our defined value ($\frac{\pi}{36}$) then the neurite is considered unaligned with the pillars and discarded. This process is

repeated and summed for all neurite vector components (T_o) and divided by the total integrated length of all neurites (T_i) to obtain our order ratio.

Before applying this procedure we preprocessed the raw confocal image of each patch to isolate the the neuron fluorescence. In particular, we started by masking large cell bodies, such as glial cells, either by hand or using a disk shaped structuring element. We then filtered in the color space to extract the fluorescence from neuron neurites and applied a intensity threshold to filter out residual fluorescence from sources other than neurons. The resolution and quality of confocal scan is the most important factor determining the error in our alignment estimates as well as the ROI processing for total growth. Some factors are mitigated by preprocessing and denoising our image. For example, background fluorescence from other features is mitigated by our preprocessing steps. However, since we needed to apply an intensity threshold over the image, we also ignored low intensity fluorescence from neurites. Another shortcoming of our approach is that it does not correct for discontinuities in the neurites. This is not a problem when the length of each segment of a neurite is longer than the gaps. If a neurite shows up as a line of disconnected dots it's alignment will not be measured correctly as the dots have no directionality, adding to the total neurite length as noise. This error can be quantified by measuring the number of short and singleton paths relative to the total length of detected neuron.

Section C: Neuron modelling

To model neuron electrophysiology, we are applying the coupled Poisson-Nernst-Planck (PNP) equations[28,29,32] to a cylindrical axon with a radial coordinate r and an axial coordinate z. In this model we apply a quasi-DC approximation where the effect of a time varying vector potential is

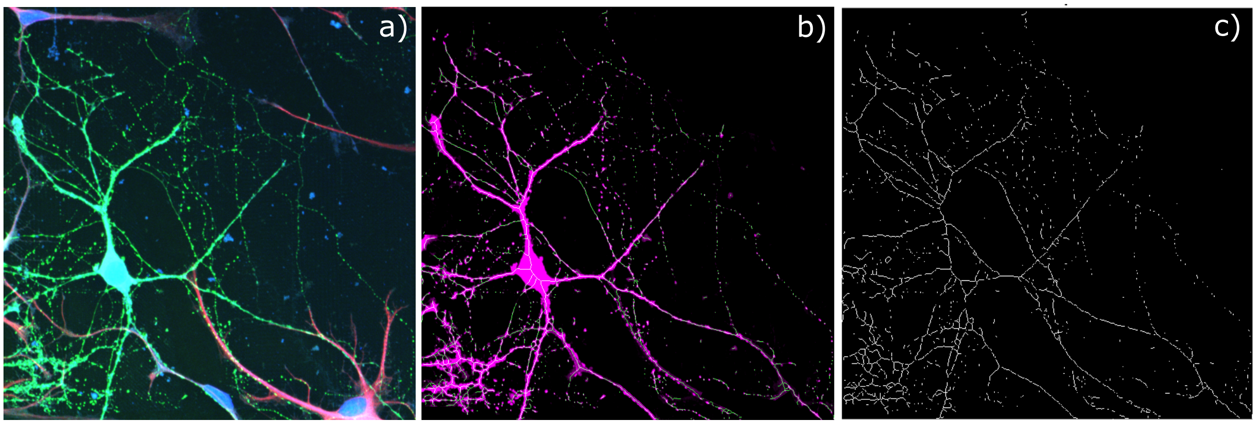

S 3 Example Images of the skeletonization process. a) The neuron confocal image, is processed to remove glia fluorescence and then 'skeletonized', b) where each neurite has a line drawn over it. The result is c) a list of lines which can be integrated to quantify neuron lengths

negligible:

$$\epsilon \nabla^2 V(r,z) = -\rho(r,z) = -e \sum_{i=1}^{M} z_i c_i(r,z) \tag{6}$$

$$\frac{\partial c_i(r,z)}{\partial t} = -\vec{\nabla} \cdot [D_i(\vec{\nabla} c_i(r,z) + \frac{1}{k_b T} z_i e c_i(r,z) \vec{\nabla} V(r,z))] \tag{7}$$

the Poisson equation (6) utilises the charged ion concentration to solve for the potential and the Nernst Planck equation (7) utilises the electric potential to model ion concentrations in terms of the electrostatic and chemical forces that act on them. In this model, $c_i(r,z)$ is the ionic concentration which is proportional to its charge density $\rho(r,z)$, and $V(r,z)$ is the electric potential, k_b is the Boltzmann constant, T the temperature, e the electric charge, D_i is the diffusion constant and z_i the ion valency. The increment i denotes which ionic species is being studied (e.g. sodium or potassium), so the total potential will be the solution to the coupled equation, summed over all the participating ion species (up to the total, M). Equations 6 and 7 are very difficult to solve even in cylindrical case where the azimuthal coordinate can be neglected due to symmetry. Thus we will

apply the two key approximations to PNP model in order to make solutions possible: reducing the number of participating ions and a travelling wave approximation. These approximations therefore also play a role in the derivation of the membrane boundary conditions.

The PNP model can in principle solve for any arbitrary number of ionic species, however, this is computationally difficult to achieve, so an approximation is made on the number of contributing species. Although there are many ions contributing to the electromagnetics of a neuron, the concentrations of many of them are small enough to be neglected such that we only consider four monovalent species in our model: sodium (Na^+), potassium (K^+), chlorine (Cl^-) and charged proteins produced by the cell (OA^-)[12,29] (see table 3). These ions are considered individually when calculating the resting charge density and the HH solution, and are averaged when calculating the PNP solution such that the positive ion concentration is given by: $c_+ = \frac{Na^+ + K^+}{2}$ and the negative ions concentration is given by: $c_- = \frac{Cl^+ + OA^+}{2}$. As these ions are monovalent, their effect on the PNP solution should be the same, allowing us to re-write the PNP equations in the following way:

$$\vec{f}_\pm(r,z) = -D_\pm \vec{\nabla} c_\pm(r,z) \mp \mu_\pm c_\pm(r,z) \vec{\nabla} V(r,z) \tag{8}$$

$$\rho(r,z) = e(c_+(r,z) - c_-(r,z)) \tag{9}$$

$$\vec{J}(r,z) = e(\vec{f}_+(r,z) - \vec{f}_-(r,z)) \tag{10}$$

where $\vec{f}_\pm(r,z)$ is the flux of the positive or negative ion species, $c_\pm(r,z)$ is the concentration

of the positive or negative ion species, $\mu_\pm$ is the ion mobility ($\mu_\pm = \frac{D_\pm e}{k_b T}$) and $\vec{J}(r,z)$ is the current density.

We then assume that the AP is a travelling wave with constant velocity. This assumption is well verified in literature as any non-linearity that disrupts the travelling wave is small enough to be insignificant.[39] This allows us to make the following change of variables using the chain rule, removing the temporal dimension and reducing the problem to a 2D spatial one:

$$\begin{aligned}\xi &= z - vt \\ \frac{\partial}{\partial t} &= \frac{\partial \xi}{\partial t}\frac{\partial}{\partial \xi} = -v\frac{\partial}{\partial \xi} \\ \frac{\partial}{\partial z} &= \frac{\partial \xi}{\partial z}\frac{\partial}{\partial \xi} = \frac{\partial}{\partial \xi}\end{aligned} \tag{11}$$

In order to solve the PNP equations in this formalism we require explicit boundary condition for both the electric potential and the ion concentrations far from the neuron as well as on the external surface of membrane. In this model, we only solve the PNP equations external to the neuron, this is reflected in our choice of boundaries. The remainder of this section will focus on the derivation of these equations.

$$V(r,\xi)|_{r\longrightarrow\infty} = 0 \tag{12}$$

$$c_\pm(r,\xi)|_{r\longrightarrow\infty} = c_{b\pm} \tag{13}$$

$$\frac{\partial V(r,\xi)}{\partial \xi}|_{\xi\to\infty} = 0 \tag{14}$$

$$f_{\pm}(r,\xi)|_{\xi\to\infty} = 0 \tag{15}$$

where we expect the potential to be zero far from the neuron radially as the ions are in an electroneutral equilibrium. The term $c_{b\pm}$ is the sum of the bulk ion concentrations for the positive and negative ions respectively which will sum to a zero charge density outside the neuron (see table 3). Axially, far from the AP along the neuron, we expect the ion concentrations and the electric potential to reach a constant equilibrium corresponding to the resting potential (-68 mV), thus the derivative of the axial potential and flux must be zero in this region.

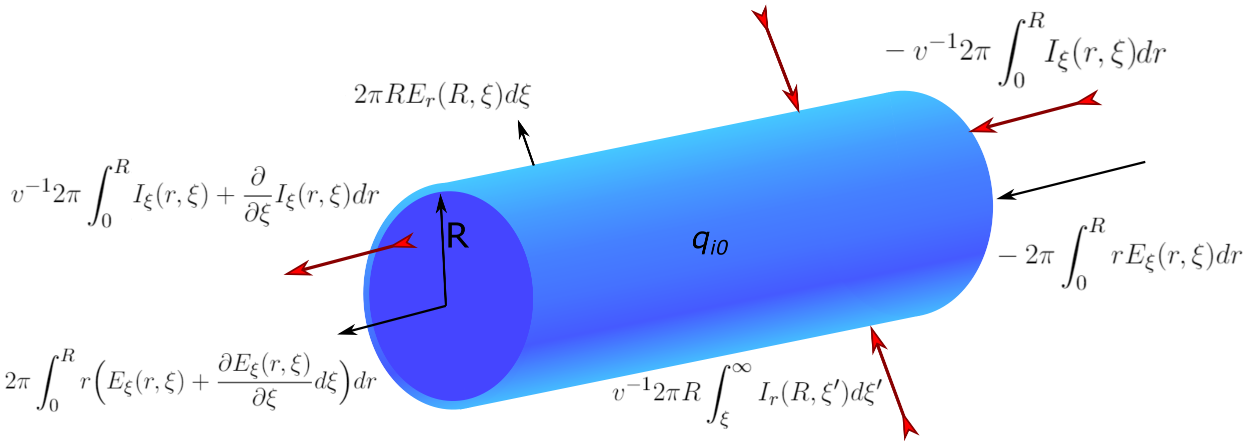

S 4 Plot of the Gaussian cylinder used in the calculations for the electric field at the membrane. The Gaussian cylinder is co-axial with the neuron with a radius R. The black arrows show the direction of the electric field components which are annotated by terms from the left hand side of Gauss' equation 16. The red arrows show the direction of the current flow which are annotated from the right hand terms of the continuity equation 20. Note the radial currents are counter propagating on opposite sides of the cylinder. The internal resting charge from equation 20 is also shown.

At the membrane, the value for the potential and ion concentrations are less clear as they are dependant on the current flowing in and out of the cell during an action potential. In order to solve

for these values we apply Gauss' law which can be simplified in the following steps:

$$\oint \vec{E}(r,\xi)\cdot dA = \frac{Q_i(\xi)d\xi}{\epsilon_r}$$
$$\rightarrow 2\pi R E_r(R,\xi)d\xi + 2\pi\int_0^R r\Big(E_\xi(r,\xi) + \frac{\partial E_\xi(r,\xi)}{\partial \xi}d\xi\Big)dr - 2\pi\int_0^R rE_\xi(r,\xi)dr = \frac{Q_i(\xi)d\xi}{\epsilon_r}$$
$$\rightarrow 2\pi R E_r(R,\xi)d\xi + 2\pi d\xi\int_0^R r\frac{\partial E_\xi(r,\xi)}{\partial \xi}dr = \frac{Q_i(\xi)d\xi}{\epsilon_r} \tag{16}$$

where R is the radius of the Gaussian cylinder coaxial to the neuron which is the same as the radius of the axon (to the outer surface of the membrane). The first term on the left hand side of equation 16 is the integral for the electric field of the length of the Gaussian cylinder, and the second term is the solution for the electric field at end-caps of the cylinder (see figure 4). In the equations, $\epsilon_r = \epsilon_0\epsilon_w$ is the absolute permittivity of the medium made up of the relative permittivity of water (ϵ_w) multiplied by the absolute permittivity of free space (ϵ_0). The terms $E_r(r,\xi)$ and $E_\xi(r,\xi)$ are the radial and axial electric fields respectively and $Q_i(\xi)$ is the internal charge per axial unit length inside the neuron. Applying the relation $\vec{E} = -\vec{\nabla}V$, the above can be rewritten in terms of the electric potential:

$$\frac{\partial V(r,\xi)}{\partial r}|_{r\rightarrow R} = -\frac{1}{R}\int_0^R r\frac{\partial^2 V(r,\xi)}{\partial \xi^2}dr - \frac{Q_i(\xi)}{2\pi R\epsilon_r} \tag{17}$$

To simplify the integral in equation 17 we will make the following substitution:

$$\frac{1}{R}\int_0^R r\frac{\partial^2 V(r,\xi)}{\partial \xi^2}dr = \frac{-\gamma R}{2}\frac{\partial^2 V(R,\xi)}{\partial \xi^2} \tag{18}$$

where γ is a constant factor which describes the divergence of the of the axial electric field (as a function of r) from a radially uniform electric field. To effectively approximate γ we consider two extreme cases for the distribution of charge inside the neuron: the resting case and the peak of an AP. In the resting case near the membrane, the charge density (and the electric field) increases dramatically forming a Debye layer.[12,40] In this case the derivative of the axial electric field will be much larger than the integrated derivative of the axial electric field and γ will have to be very small in order to compensate ($\gamma \to 0$). This makes physical sense as in the resting condition we expect the change in the axial electric field to be negligible. In the other extreme case, an AP will cause a depolarizing influx of charge which will cause the interior charge density to become more radially uniform.[29] In this case the derivative of the electric field will become radially constant and γ will become one. Thus, throughout the course of the neuron going from the resting condition, into the peak of an AP and returning to the resting condition the value of γ must fall between zero and one. However even at its highest value, ($\gamma = 1$), this term has a negligible contribution to the overall electric field. This is due to the charge per unit length inside the neuron is mostly uniform across the length scales we use (microns), making the net surface integral of the end caps negligible similar to a line of charge.

The second term in equation 17 requires knowledge about the internal charge in the neuron, $Q_i(\xi)$. We can derive this quantity via the continuity equation for charge. The total internal charge is made up of the charge during the neuron resting condition plus the currents moving charge in

and out of the neuron due to an AP:

$$
\begin{aligned}
Q_i(\xi) = q_{i0}(\xi) - v^{-1}2\pi \int_0^R \int_\xi^\infty \Big(I_\xi(r,\xi) + \frac{\partial}{\partial \xi} I_\xi(r,\xi) - I_\xi(r,\xi)\Big) r \, dr d\xi - v^{-1}2\pi R \int_\xi^\infty I_r(R,\xi')d\xi' \\
\rightarrow Q_i(\xi) = q_{i0}(\xi) - v^{-1}2\pi \int_0^R \int_\xi^\infty \frac{\partial}{\partial \xi} I_\xi(r,\xi) r \, dr d\xi - v^{-1}2\pi R \int_\xi^\infty I_r(R,\xi')d\xi' \\
\rightarrow Q_i(\xi) = q_{i0}(\xi) - v^{-1}2\pi \int_0^R I_\xi(r,\xi) r \, dr - v^{-1}2\pi R \int_\xi^\infty I_r(R,\xi')d\xi'
\end{aligned}
\tag{19}
$$

in this equation, $q_{i0}(\xi)$ is the total charge per unit length in the neuron resting condition, that is, the charge existing in the cell in the absence of an AP. The AP then alters the total charge by introducing radial membrane ($I_r(R,\xi)$) and internal axial ($I_\xi(r,\xi)$) currents which are currents per unit area. We then apply Ohm's law to turn the axial current into the integral of the axial electric field.

$$
Q_i(\xi) = q_{i0}(\xi) + v^{-1}2\pi \int_0^R \sigma_{i0} E_\xi(r,\xi) r \, dr - v^{-1}2\pi R \int_\xi^\infty I_r(R,\xi')d\xi' \tag{20}
$$

Similar to the approximation we made in equation 18, we can re-express the second term on the right hand side of equation 20 by removing the integral and introducing a constant factor η, to represent the deviation from a uniform potential and conductivity. This results in a quasi-Ohm's law approximation where σ_{i0} is the total internal conductivity.

$$
Q_i(\xi) \approx q_{i0}(\xi) - \eta\pi R^2 \sigma_{i0} v^{-1} \frac{\partial V(R,\xi)}{\partial \xi} - v^{-1}2\pi R \int_\xi^\infty I_r(R,\xi')d\xi' \tag{21}
$$

Approximating η is a little more difficult than γ as we need to consider the conductivity as

well as the electric field. The conductivity is a function of the total concentration of ions which we know will change with radius due to the Debye layer. During the peak of an AP, we expect the ion concentration (and conductivity) to be mostly uniform along with the axial electric field so we will set η to one. During the resting case, the conductivity will be mostly uniform with an increase at the Debye layer where the concentrations increase. However, increases to the concentration in the Debye layer have to be dramatic to have an effect on the overall fields due to the fact that the Debye layer is a very small region of charge compared to the overall volume of the interior neuron. For this reason we will approximate η to one for all times during an AP.

Substituting equation 21 into equation 17 gives the following:

$$\frac{\partial V(r,\xi)}{\partial r}|_{r=R} = -\frac{q_{i0}(\xi)}{2\pi R\epsilon_r}+\frac{1}{2\pi R\epsilon_r}\Big(\eta\pi R^2\sigma_{i0}v^{-1}\frac{\partial V(R,\xi)}{\partial \xi}+v^{-1}2\pi R\int_{\xi}^{\infty} I_r(R,\xi)d\xi'\Big)-\frac{\gamma R}{2}\frac{\partial^2 V(R,\xi)}{\partial \xi^2} \tag{22}$$

this equation requires the radial membrane current as well as the axial derivative of the electric potential. To find these quantities we apply the Hodgkin-Huxley (HH) equations.[29,31,38] The HH equations are a well known and experimentally verified set of coupled equations which describe the transmembrane potential, $V_m(t)$, and radial membrane currents in terms of the ion flow across the membrane mediated by ion channels opening and closing. Note that in our model, the neuron isn't clamped so we will need to consider an axial current as well:

$$\frac{\partial V_m(t,z)}{\partial t} = \frac{1}{C_m}\Big(I_r(R,t)+\frac{1}{R}\int_0^R \frac{\partial I_\xi(r,z,t)}{\partial z}rdr\Big) \tag{23}$$

$$I_r(R,t) = \Big(- I_d(R,t) - g_{Na} m(t)^3 h(t)(V_m(t) - V_{Na}) - g_{NaL}(V_m(t) - V_{Na}) \\ - g_K n(t)^4 (V_m(t) - V_K) - g_{KL}(V_m(t) - V_K) \Big) \tag{24}$$

$$\frac{dm}{dt} = \phi(\alpha_m(t)\big(1 - m(t)\big) - \beta_m(t) m(t)) \tag{25}$$

$$\frac{dh}{dt} = \phi(\alpha_h(t)\big(1 - h(t)\big) - \beta_h(t) h(t)) \tag{26}$$

$$\frac{dn}{dt} = \phi(\alpha_n(t)\big(1 - n(t)\big) - \beta_n(t) n(t)) \tag{27}$$

where C_m is the membrane capacitance, g_x is the conductance for a particular ion species, g_{xL} is the leak conductance of the same species V_x is the Nernst potential of an ion species, ϕ is the ion channel time constant, $I_d(R,t)$ is an input current (e.g. a presynaptic potential), m, n, and h are parameters which describe the opening and closing of ion channels which are dependant on the factors α_x and β_x. The α_x and β_x terms are found through experimental fitting which in this calculation is taken from Zandt et al.[38] As the AP wavelength is orders of magnitude larger than the radius of the neuron,[12] we expect the integrated derivative of the axial current to be much smaller than the radial current as the axial current will change slowly over the long wavelength of the AP. Therefore, the second term on the right hand side of equation 23 can be neglected.

We relate the HH equations to the terms in equation 22 by making the following substitutions

from the travelling wave approximation:

$$\frac{\partial V_m(t)}{\partial t} = \frac{-1}{C} I_r(t) \rightarrow \frac{\partial V(R,\xi')}{\partial \xi} = \frac{v^{-1}}{C} I_r(R,\xi) \tag{28}$$

$$\int_t^\infty \frac{\partial V(R,t')}{\partial t'} dt' \rightarrow -v \int_\xi^\infty \frac{\partial V(R,\xi)}{\partial \xi'} d\xi' = -v\big(V(R,\infty) - V(R,\xi)\big) = \frac{-1}{C} \int_\xi^\infty I_r(R,\xi') d\xi' \tag{29}$$

where $V(R,\infty)$ is equivalent the resting potential V_{rest}. Equations 28 and 29 relates the HH transmembrane potential and radial current to $V(R,\xi)$ and $I_r(R,\xi)$ in equation 22 which we derived from Gauss' law. In this formalism, we describe $V(R,\xi)$ as the cross-sectional average of the potential which is effectively the transmembrane potential. This allows us to substitute these terms into equation 22 to obtain a simpler boundary condition whose potential and current terms can be added in from the HH solutions. We can also use the HH current solution to calculate the ion flux which can be used as a membrane concentration boundary condition. Putting all the equations together gives the membrane boundary conditions for the membrane electric fields as well as the ion flux:

$$-E_r(R,\xi) = -\frac{q_{i0(\xi)}}{2\pi R\epsilon_r} + \frac{1}{2\pi R\epsilon_r}\Big(\eta\pi R^2\sigma_{i0}v^{-2}C^{-1}I_r(R,\xi) + 2\pi RC\big(V_{rest} - V(R,\xi)\big)\Big) - \frac{\gamma R v^{-1}}{2C}\frac{\partial I_r(R,\xi)}{\partial \xi} \tag{30}$$

$$\frac{\partial c_+(r,\xi)}{\partial r}\Big|_{r=R} = \frac{N_A}{e} 2\pi R I_r(R,\xi) \tag{31}$$

$$\frac{\partial c_-(r,\xi)}{\partial r}|_{r=R} = 0 \tag{32}$$

where N_A is Avogadro's constant. Equation 31 simply states that the rate of change in the positive ion concentration is directly proportional to the radial current. Due to the assumption that there is only positive ion flow across the membrane,[29,38] the negative ion flux is zero.

To obtain the magnetic field boundary conditions we apply Ampere's laws where the axial current in the membrane boundary condition is treated in the same way as equation 21.

$$\vec{\nabla} \times \vec{B}(r,\xi) = \mu_0 \vec{J}(r,\xi) \tag{33}$$

where the external current density is given from equation 10 and the effect of a polarizing current is negligible (quasi-DC approximation). The boundary conditions are as follows:

$$\vec{B}(r \to \infty) = 0 \tag{34}$$

$$\vec{B}(\xi \to \infty) = 0 \tag{35}$$

the current densities are zero far from the AP axially and radially, so the magnetic field will be zero. At the membrane, the magnetic field is dependent on the axial and radial currents (I_ξ and I_r respectively). However, the radial current contribution to the magnetic field can be considered negligible as each radial current component on the cylinder would be cancelled out by the radial current component on the opposite side of the cylinder (see figure 1). This assumption is well

established in literature[7] and means that the magnetic field will be directly proportional to the axial current, similar to a current carrying wire:

$$\vec{B}_r(r,\xi)|_{r=R} = -\frac{\mu_0}{2\pi R} I_\xi(R,\xi) \tag{36}$$

the axial current can be related to the derivative of the potential from Ohms law (equation 21):

$$\vec{B}_r(r,\xi)|_{r=R} = -\frac{\mu_0}{2}\eta R\sigma_{io}\frac{\partial V(R,\xi)}{\partial \xi} \tag{37}$$

the potential derivative can be related to the radial current using the substitution equation 28:

$$\vec{B}_r(r,\xi)|_{r=R} = -\frac{\mu_0}{2}\eta R\sigma_{i0}C^{-1}v^{-1}I_r(R,\xi) \tag{38}$$

this means that despite the radial current contributing negligibly to the magnetic field, it is a driving force in the creation of the axial current which creates the magnetic field indirectly and can be derived using the HH equations.

The membrane boundary conditions derived above are all that is required to find the full electromagnetic quantities external to a neuron. Equations 30, 31 and 32 are the main results of this paper and can be used with the PNP equations to solve for the electric potential and ion concentrations. The ion concentrations can then be used to derive the external current density which can be used with equation 38 and Ampere's law to find the external magnetic fields. The parameters used in these equations are given in Table 3. Included in the table are the dimensions of the cylindrical neuron used in the simulation. Although many of the values were obtained from literature, calculations of the conductivities, mobility's and the resting potential were performed to ensure the

values were self-consistent.

Table 3 Table of Parameters used in the calculations, all other terms used (e.g. ion mobility's or Nernst potentials) are derived from these values. Values that are un-sourced were chosen by the authors to suit the model

Parameter	Description	Value	Source
K_i	Internal Potassium Concentration	155 mmol/L	Lopreore[29]
K_e	External Potassium Concentration	4 mmol/L	Lopreore[29]
Na_i	Internal Sodium Concentration	12 mmol/L	Lopreore[29]
Na_e	External Sodium Concentration	145 mmol/L	Lopreore[29]
Cl_i	Internal Chlorine Concentration	4.2 mmol/L	Lopreore[29]
Cl_e	External Chlorine Concentration	123 mmol/L	Lopreore[29]
OA_i	Internal Protein Concentration	162.802 mmol/L	Lopreore[29]
OA_e	External Protein Concentration	26 mmol/L	Lopreore[29]
g_{Na}	Total Sodium conductance	100 mS/cm^2	Zandt[38]
g_{NaL}	Sodium leak conductance	0.0175 mS/cm^2	Zandt[38]
g_K	Total Potassium conductance	40 mS/cm^2	Zandt[38]
g_{KL}	Potassium leak conductance	0.05 mS/cm^2	Zandt[38]
ϕ	HH time constant	3 ms^{-1}	Zandt[38]
C	Membrane Capacitance	1 μF/cm^2	Zandt[38]
T	Temperature	310°K	-
D_K	Potassium Diffusion coefficient	1.957×10^{-9} m^2/sec	Samson[41]
D_{Na}	Sodium Diffusion coefficient	1.334×10^{-9} m^2/sec	Samson[41]
D_{Cl}	Chlorine Diffusion coefficient	2.032×10^{-9} m^2/sec	Samson[41]
D_{OA}	Protein Diffusion coefficient	2.00×10^{-9} m^2/sec	Samson[41]
ϵ_r	Absolute Permittivity of water	$80 \times 8.854 \times 10^{-12}$ C/Vm	-
μ_K	Potassium mobility	7.328×10^{-8} m^2 s^{-1} V^{-1}	Lopreore[29]
μ_{Na}	Sodium mobility	4.995×10^{-8} m^2 s^{-1} V^{-1}	Lopreore[29]
μ_{Cl}	Chlorine mobility	-7.609×10^{-8} m^2 s^{-1} V^{-1}	Lopreore[29]
μ_{OA}	Protein mobility	-7.489×10^{-8} m^2 s^{-1} V^{-1}	Lopreore[29]
V_{rest}	Resting Potential	-68 mV	Zandt[38]
R_n	Radius of neuron	500 nm	Liewald[17]
L_n	axial length of neuron	2 mm	-
R_n	Radius nano-mesh	10 nm	-
R_n	Radius of external solution	1.5 μm	-

Figure 5 is the result of the membrane boundary condition derivations. Figure 5a) is the membrane electric field solution from equation 30, figure 5b) is the membrane magnetic field from equation 38 and figure 5c) is the membrane positive ion flux taken from equation 31, which is simply the radial current, placed in units of ions per metre squared. These plots form the orange lines used for reference on figure 3 of the main paper.

Figure 6 shows the results of the HH equations. Figure 6a) is the transmembrane potential from equation 23, figure 6b) is the solutions to the gating parameters in equations 25, 26, and 27. Figure 6c) is the membrane radial current solution to equation 24. As expected, these solutions match well to HH equations presented in other works[29,31,38] which help validate the membrane electric and magnetic field solutions we derive in this manuscript.

Section D: Electric fields in diamond pillars

Consider a geometry where a neuron runs over a single cylindrical diamond pillar where the tip of the pillar is in full contact with the neuron (figure 7). Assume that the charge inside the neuron is unperturbed by the presence of the pillar and that Debye screening fixes the electric potential on the sidewalls of the pillar to be zero. Note that this ignores the small region close to the neuron (i.e. within the Debye layer) where the potential is non-zero on the sidewalls. We expect these assumptions to be good as long as diameter of the pillar isn't so large that affects the function of the neuron, but sufficiently large compared to the Debye length (1 nm) such that the non-zero potential within the Debye layer has negligible influence on the electric field in the region of the pillar's central axis. This is where it is desirable to implant the NV centers. Given these assumptions and adopting the local cylindrical coordinate system of the pillar depicted in figure 7, Laplace's equation yields the following electric potential within the nanopillar:

$$V(r,z) = \left(\frac{d}{k}\right) E_m J_0\left(\frac{rk}{(d/2)}\right) e^{(-k/(d/2))(z-R_{mem})} \tag{39}$$

where J_0 is the zeroth Bessel function, d is the diameter of the diamond pillar (200 nm), R_{mem} is the radius of the neurite (500 nm) and $k \approx 2.4$, the first zero solution of the Bessel function. In addition there is E_m, the membrane electric field, i.e. the electric field at $z = R_{mem} = 500$ nm,

the membrane boundary. This value is calculated from equation 30 with the same parameters as used in table 3 but with a diamond permittivity ($\epsilon \approx 6$) instead of water. This yields a membrane electric field of 4.54×10^{10} mV/m. It then follows from $\vec{E} = -\vec{\nabla} V$, that the axial electric field inside the pillar is:

$$E_z = E_m J_0\left(\frac{rk}{d/2}\right) e^{(-k/(d/2))(z - R_{mem})} \tag{40}$$

On the central axis of the pillar where $r = 0$, the Bessel function becomes 1 and the electric field propagation becomes:

$$E_z = E_m e^{(-k/(d/2))(z - R_{mem})} \tag{41}$$

As mentioned in the main text, the axial field decays exponentially from the tip with a decay constant of $k/(d/2)$.

Figure 8 depicts the a different modelled geometry, where the neurite runs along the side of the pillar towards the top. In this model, the surface of the pillar which is in contact with the neurite is the same area as in the case with the neurite running on top of the pillar. This contact area is considered small compared to the overall surface area of the enclosing cylinder segment. This small contact area is the basis for the assumption that the diamond pillar contact won't affect the natural function of the neuron. The contact area has the same surface electric field from the neuron as the previous case (4.54×10^{10} mV/m) and the rest of the pillar has its potential fixed at zero. With these boundary conditions the electric field propagation inside the neuron can be solved numerically using COMSOL Multiphysics. The solution in this geometry is shown as a 2D slice density plot in figure 9.

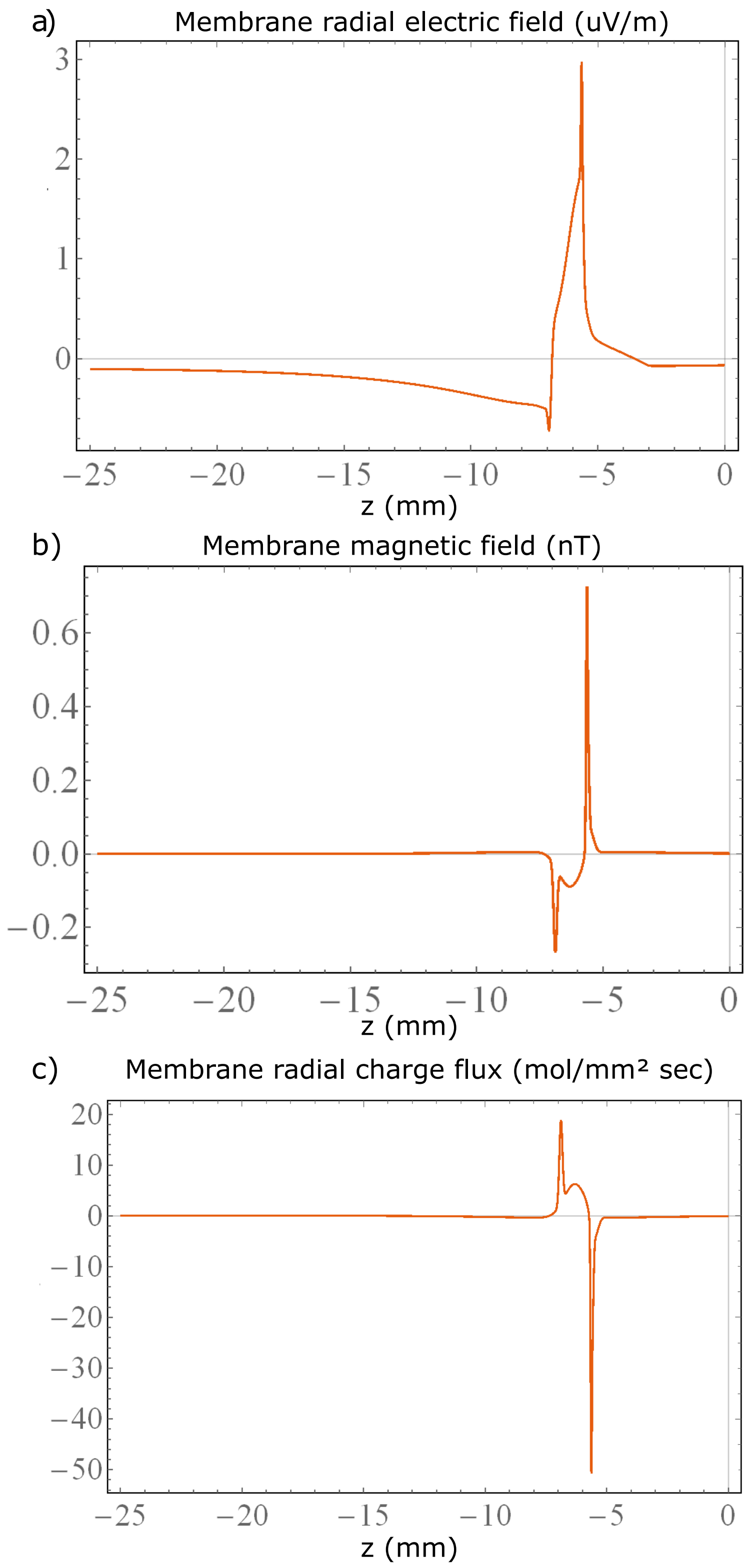

S 5 Plots for the a) membrane electric field, b) membrane magnetic field. The plots were derived from equations 30 and 38 respectively. c) Plot of the membrane flux derived from the HH equations. All three of these plots were used as the orange sketch lines in the 2D surface plots of figure 3 of the main paper.

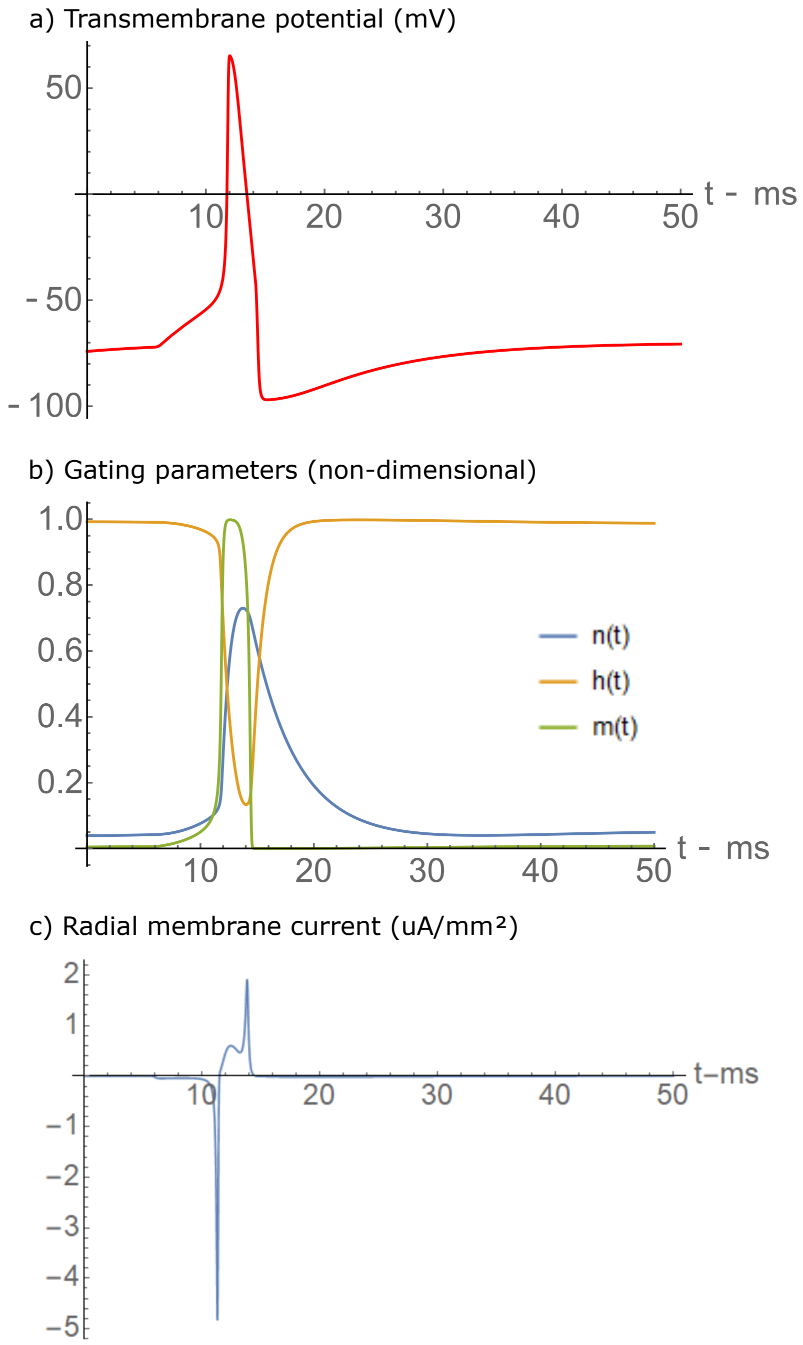

S 6 Plots of the a) Hodgkin-Huxley solutions (equation 23) for the potential, b) gating parameters (equations 25, 26, 27) and c) the radial current derived from equation 24.

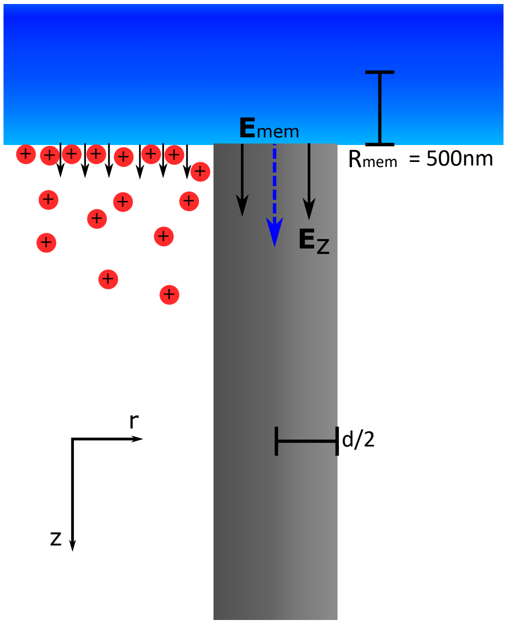

S 7 Image of the geometry considered for the electric field inside the diamond when the neurite (blue) runs over the top of diamond pillar (grey). The image features the positive ions forming the Debye layer outside the neuron which doesn't exist inside the diamond as well as the coordinate system used to obtain the solution. The blue dashed line represents the 1D solution used in figure 4b of the main text.

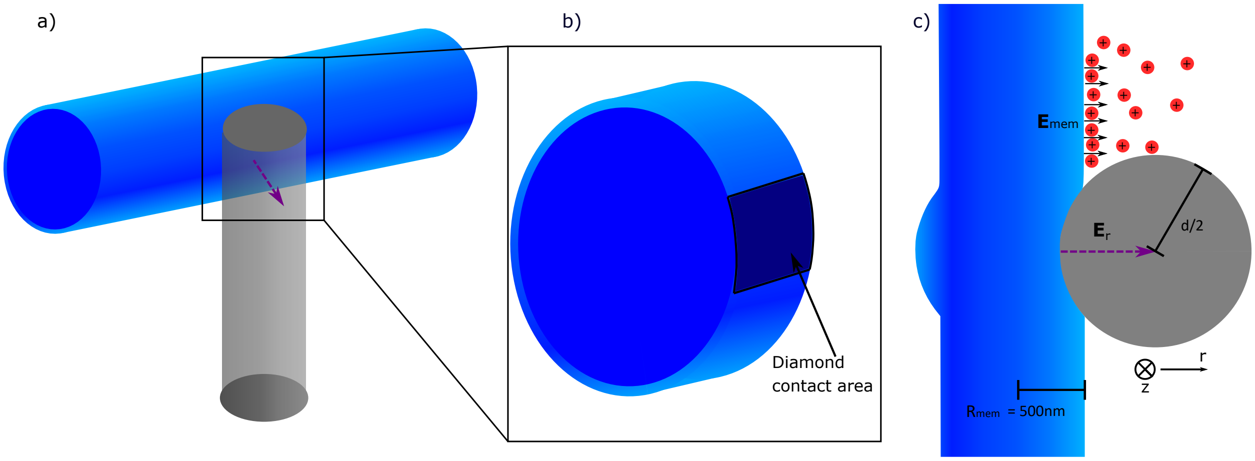

S 8 Image of the geometry considered for the electric field inside the diamond when the neurite (blue) runs across the side of diamond (grey) towards the tip of the pillar. a) The diamond pillar the neurite makes contact towards the top of the pillar and the cutout of the neurite segment b) shows a dark shaded area where the pillar makes contact with the neuron segment. The contact area is small compared to the overall surface area of the enclosing cylinder segment. c) The top down view of the same system in a)/b). The image features the positive ions forming the Debye layer outside the neuron which doesn't exist inside the diamond as well as the coordinate system used to obtain the solution. The purple dashed line represents the 1D solution used in figure 4b of the main text.

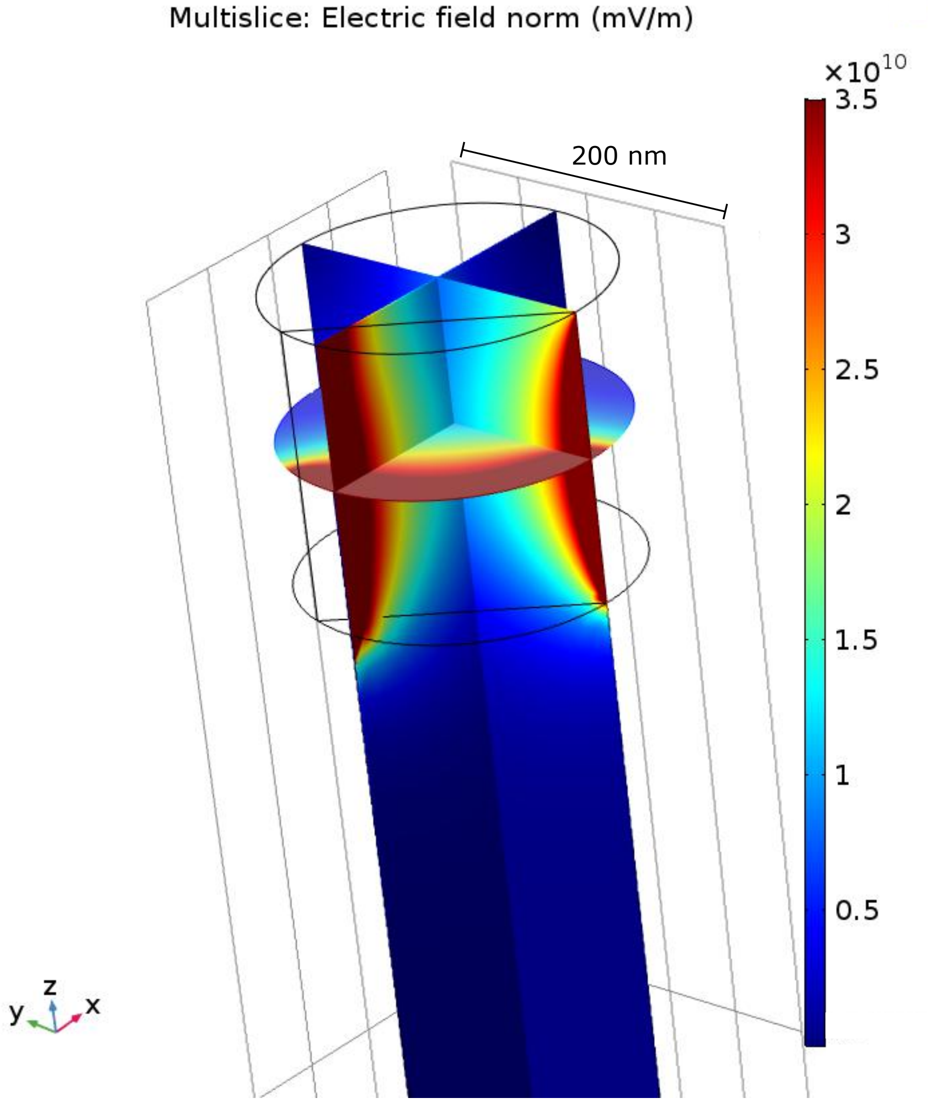

S 9 Solution for the electric field with a neurite in contact with the side of the pillar tip. The geometry with the neurite is shown in figure 8. The neurite contact area is marked by the surface of the tip cut out by the black wire-frame rectangle.
